# Simulation of *Fe*_3_*O*_4_ Nanoparticle Transport in a Diseased Curved Artery Under Thermal Influence: Implications for Targeted Drug Delivery

**DOI:** 10.3390/nano16110677

**Published:** 2026-05-28

**Authors:** Poonam, Bhupendra K. Sharma, Rishu Gandhi, David Laroze

**Affiliations:** 1 Department of Mathematics, Birla Institute of Technology and Science Pilani, Pilani 333031, India; aggarwalpurnima123@gmail.com; 2 Department of Mathematics, Applied Science Cluster, University of Petroleum and Energy Studies, Dehradun 248007, India; 3 Instituto de Alta Investigación, Universidad de Tarapacá, Casilla 7D, Arica 1000000, Chile

**Keywords:** hematocrit-dependent viscosity, stenosis, aneurysm, heat transmission, curved arteries, tailored drug administration

## Abstract

This study examines non-Newtonian electromagnetohydrodynamic (EMHD) blood flow via a diseased curved artery with minor stenosis and an aneurysm, adding a no-slip boundary condition, using targeted medication delivery of nanoparticles. The non-Newtonian behavior of blood flow is accounted for by the Casson fluid model. Using Corcione’s model, we have calculated the effective viscosity and thermal conductivity of nanofluids. The interaction of the nanofluid with physical phenomena such as viscous dissipation, electro-osmosis, radially applied uniform magnetic field and Joule heating can change the hemodynamic parameters of the fluid. The Crank–Nicolson approach has been used to calculate the velocity, temperature, and concentration patterns within the Debye–Huckel linearization approximation. Streamlines are delineated to analyze flow patterns across distinct physical factors. This study supports the design of magnetically guided $Fe_{3}O_{4}$ nanoparticle–based targeted drug delivery systems for treating vascular diseases such as stenosis and aneurysm, improving site-specific therapeutic efficiency. The numerical insights into thermal effects and arterial geometry help to optimize nanoparticle transport, enhancing treatment precision while minimizing systemic side effects.

## 1. Introduction

Cardiovascular disease is a serious public health concern worldwide, with numerous interconnected factors leading to its development. Acknowledging these factors and applying lifestyle modifications can help to minimize the risk and severity of CVD. Regular medical treatment and proactive control are essential for those who are at higher risk or have already been diagnosed with cardiovascular disease. There are some factors that are responsible for all these cardiovascular diseases like genetics, unhealthy lifestyle, hypertension, high cholesterol which leads to plaque formation in arteries (atherosclerosis), obesity (a myocardial infarction), heart attack, irregular heartbeats, heart failure, PAD or peripheral artery disease and blood vessel wall bulging (aneurysm) that has the potential to burst and cause catastrophic bleeding. Sinha et al. [1] conducted a theoretical investigation on the peristaltic transport of MHD blood flow with variable viscosity in an asymmetric capillary blood vessel imposing velocity and thermal slip conditions. Akbar et al. [2] provided a mathematical model for simulating steady-state viscous blood flow, including copper nanoparticles through a composite stenosed artery with permeable walls, which includes wall slip hydrodynamic and thermal buoyancy effects. The artery is modeled as an isotropic elastic tube with a variable viscosity formulation for the flowing blood. Kumawat et al. [3] conducted an investigation into the hemodynamics within a curved stenosed artery, employing a two-phase fluid model characterized by variable viscosities in both constituent phases. They posited that viscosity fluctuates with hematocrit levels within the core region and temperature variations in the plasma region. In their study, they scrutinized both Newtonian and non-Newtonian fluid models for the oxygenated and deoxygenated blood. Gandhi and Sharma [4] investigated the dynamics of hybrid nano-blood flow through an anatomically compromised vertically oriented artery with implications for drug delivery applications. Hussain et al. [5] evaluated the qualitative aspects of blood flow and the degree of obstruction engendered by diverse arterial pathway geometries through the application of sophisticated computational fluid dynamics software. Their findings indicated that the geometric configuration of the stenosis markedly affects the temperature, velocity, and comprehensive pressure reduction within the constriction zone of the artery. Sharma et al. [6] conducted an investigation into the magnetohydrodynamic hybrid nanofluid flow within an inclined stenotic artery, taking into account the dependency of blood viscosity on hematocrit levels.

Aneurysms represent a subject of extensive scholarly investigation owing to their complex characteristics, with research efforts predominantly focused on various determinants that influence their behavior, particularly concerning the processes of formation, progression, and rupture of aneurysms. This encompasses clinical investigations, experimental fluid dynamics, as well as computational and numerical analyses that facilitate a comprehension of aneurysm dynamics via patient-specific models and flow conditions. Clinical inquiries have been particularly geared towards delineating critical morphological phases associated with aneurysm genesis and rupture. The analysis reveals that the dimensions of aneurysms in male patients average 9.2 mm, in contrast to 7.4 mm in female patients [7,8,9]. Preliminary studies on aneurysms conducted by Ferguson [10] suggested that turbulence and intra-aneurysmal pressure may contribute to the weakening and expansion of the aneurysmal wall, thereby elevating the rupture risk. Subsequently, it was posited that aneurysms might evolve as a result of hemodynamically stressed degenerative lesions [11] or due to high-flow fluctuations that could facilitate either growth or rupture [12]. Researchers have leveraged computational methodologies to simulate a variety of aneurysm flow conditions and to construct authentic aneurysm geometries derived from MRI imaging of individual patients [13,14,15,16,17].

Nanofluids (NFs) have garnered substantial scholarly attention within the realm of biomedical engineering in recent years. Among various related disciplines, NFs exhibit considerable utility within the biomedical sector, particularly in the domains of drug delivery, lithotripsy, wound healing, management of lung cancer, treatment of pulmonary tuberculosis, therapy for brain tumors, and innovative approaches such as nano cryosurgery. A nanofluid is defined as a fluid that incorporates suspended nanoparticles (NPs) within a base fluid, which may include water or oil. The dimensions of NPs typically range from 1 to 100 nm, and their incorporation can markedly influence the physicochemical properties of the fluid. These nanoparticles manifest in diverse geometries, encompassing cylinders, blades, films, wires, tubes, fibers, spherical particles, rods, shells, and various polymeric configurations, among others. Beyond their extensive diagnostic applications, NFs provide efficacious therapeutic interventions for cardiovascular ailments. The suspension of NPs within a base fluid to enhance the thermophysical properties is referred to as “nanofluid,” as articulated by Choi and Eastman [18]. Generally, these materials consist of carbides, metals, or oxides characterized by elevated thermal conductivity. Gandhi et al. [19] elucidated the influence of hybrid nanoparticles on hemodynamics within a porous cylindrical artery exhibiting stenosis when subjected to an external magnetic field. Recent investigations have underscored the increasing significance of multifunctional nanoparticles and bio-inspired composite materials within the realm of biomedical applications. Deng et al. [20] engineered multifunctional nanoparticles specifically for the therapeutic management of rheumatoid arthritis through a precision-targeted nanotherapeutic strategy. Their approach encompassed the design of nanoparticles endowed with regulated drug delivery capabilities coupled with anti-inflammatory effects. The findings from the study indicated an enhancement in therapeutic efficacy alongside a reduction in inflammatory responses within preclinical models; however, the long-term biocompatibility and clinical viability of the proposed system remain ambiguous. Dong et al. [21] proposed a copper-based multimodal drug delivery platform aimed at augmenting therapeutic efficacy. The authors utilized copper-infused nanomaterials to facilitate concurrent drug delivery and therapeutic monitoring. Their results revealed an improvement in drug release kinetics and enhanced treatment effectiveness, notwithstanding the identification of cytotoxicity and long-term safety concerns regarding metal-based nanoparticles as significant limitations. Xiang et al. [22] synthesized gelatin-based artificial vascular grafts employing bio-inspired composite fabrication methodologies. The resultant grafts demonstrated commendable mechanical properties, flexibility, and biocompatibility, rendering them suitable for applications in vascular tissue engineering. Nevertheless, the study acknowledged limitations pertaining to long-term durability and a lack of sufficient in vivo validation under physiological conditions. Kanwar et al. [23] examined 3D composite scaffolds for bone tissue regeneration via advanced scaffold fabrication techniques and biomaterial integration strategies. Their results indicated improvements in osteogenic differentiation, cell adhesion, and the potential for tissue regeneration. However, challenges associated with large-scale manufacturing, the complexity of fabrication processes, and production costs were not comprehensively addressed. Wang et al. [24] presented an acid-responsive therapeutic system targeting cartilage degeneration. The methodology was predicated on a pH-sensitive material capable of facilitating targeted therapeutic release in degenerative environments. Experimental findings demonstrated enhanced protection of cartilage and improved regenerative outcomes; however, the study was constrained by the absence of extensive long-term and clinical-scale evaluations. These investigations collectively illustrate the diverse applications of nanotechnology-based therapeutic and regenerative systems while concurrently emphasizing enduring challenges related to biocompatibility, scalability, long-term stability, and clinical translation.

The magnetic characteristics exhibited by blood can be attributed to the presence of hemoglobin, an iron-containing protein located within erythrocytes. Due to the intricate interactions among the intracellular protein, hemoglobin, and the cellular membrane, blood manifests properties akin to those of a magnetic fluid. The application of magnet therapy has been a component of medical treatments for numerous years. It is beneficial in alleviating intense pain, enhancing oxygenation, promoting the regeneration of bone and tissue, and improving blood circulation, among other advantages. Positively charged ions exist within the bloodstream, mirroring the ionic composition found in other tissues and cellular structures. When subjected to a magnetic field, the flow of blood is influenced, compelling the movement of ionic particles by the principles outlined by the Lorentz force law. The ionic constituents within the blood vessels exhibit oscillatory motion induced by the applied magnetic field, thereby generating a current within the bloodstream. Consequently, the electromotive force, alterations in ionic configurations, and the resultant currents instigate a modification in the flow field. The incorporation of iron oxides within hemoglobin molecules engenders magnetic fields that can either repel or attract blood. The most commonly utilized magnetic nanoparticles include iron oxides (Fe_2_O_3_ and Fe_3_O_4_), manganese ferrite (MnFe_2_O_4_), and cobalt ferrite (CoFe_2_O_4_). Due to their biocompatibility and elevated magnetization, it has been established by Berry and Curtis [25] that magnetite (Fe_3_O_4_) nanoparticles are typically favored in magnetic drug targeting (MDT). Furlani and Furlani [26] devised a mathematical framework to predict the magnetic targeting of multifunctional carrier particles intended for the delivery of therapeutic agents to malignant tissues in vivo. Sharma et al. [27] employed mathematical modeling techniques to simulate the dynamics of magnetic nanoparticles within an artery influenced by a magnetic field, underscoring the efficacy of the model in facilitating magnetic targeting. Ketchate et al. [28] conducted a stability analysis of Casson blood flow containing hematite and magnetite, two distinct categories of magnetic nanoparticles, traversing through an anisotropic porous artery. Sharma et al. [29] investigated the implications of electromagnetohydrodynamics (EMHD), taking into account an exponential spatial and thermally dependent heat source over a nonlinear stretching surface characterized by variable thickness.

Magnetohydrodynamics (MHD) encompasses the examination of the behavior of electrically conductive fluids, such as liquid metals, plasmas, and saline solutions, in the presence of magnetic fields. The implications of MHD for nanoparticles extend to their stability, transport characteristics, and dispersion behaviors. The application of magnetic fields can significantly modify the velocity and trajectory of nanoparticle movement, either amplifying or diminishing heat transfer rates, while simultaneously enhancing the uniform distribution of nanoparticles within a base fluid. Consequently, the amalgamation of MHD principles with nanoparticle technologies heralds significant advancements in the optimization of nanofluid performance across diverse industrial and scientific domains [30,31,32,33]. Pattnaik et al. [34] scrutinized metallic nanoparticles, revealing that MHD exerts a pivotal influence on the enhancement of heat transfer in porous media. This research underscores the critical role of MHD in refining the thermal efficacy of nanofluids. Mohana and Rushi [35] investigated various nanoparticle geometries and their consequential effects on the flow dynamics and thermal transfer properties of MHD Cu–water nanofluids with integrated heat sources or sinks. Farooq et al. [36] evaluated the influence of MHD on kerosene oil infused with silver and manganese zinc ferrite nanoparticles, determining that an increase in suction, volume fraction, and Lorentz force significantly amplifies the heat transfer capabilities of the hybrid nanofluid.

Electro-osmosis-driven transport has garnered significant scholarly attention in recent years, attributed to its extensive applications in the biomedical domain. It provides a multitude of substantial applications across electromechanical, biochemical, medical, and biological sciences, among others. The phenomenon of electro-osmosis pertains to the investigation of electrolyte movement within a conduit exhibiting a charged wall, induced by the application of an external electric field. Its applications are varied and include Key areas of advanced biomedical research and healthcare innovation include the development of microdevices for biochemical analysis, drug action and interaction mechanisms, implantable devices related to vision, in vitro cell growth techniques, biomaterial frameworks for tissue engineering, medical testing and disease detection, therapeutic procedures, and health treatments nanorobotic propulsion in medical applications, as well as blood and urine diagnostics. The electro-osmosis phenomenon was initially elucidated by Reuss [37] in the year 1809. Subsequently, Wiedemann [38] provided its mathematical formulation. Since that time, numerous simulation studies have been undertaken to elucidate the flow characteristics associated with electro-osmosis-based phenomena. Although electro-osmotic flow of Newtonian and non-Newtonian fluids in microchannels and microtubes has been studied in great detail, its applicability to artery systems is still relatively unexplored. The hemodynamic behaviour of electro-magnetized hybrid nanofluid blood flow via sick arteries, driven by electro-osmotic forces, was examined by Abdelsalam et al. [39]. A related study by Das et al. [40] provided important insights into microscale flow regulation by deriving analytical solutions for the electro-osmotically induced transport of ionized, non-Newtonian hybrid nanofluids in microchannels exposed to strong magnetic fields. In a sinusoidal wavy heated tube with high zeta potential, Saleem et al. [41] examined the electric double layer (EDL)-induced flow and heat transfer of Casson fluid, emphasizing the important role of the Debye–Huckel parameter on physiological fluid dynamics. EDL-driven biofluid transport across a microchannel was theoretically analyzed by Noreen et al. [42], who found that the fluid pressure gradient decreased as the electro-osmotic parameter values increased. Similarly, employing a non-Newtonian blood model, Akhtar et al. [43] offered hemodynamic insights into electro-osmotic flow in sick arteries, showing that electro-osmotic parameters can be tuned to influence blood flow successfully. The electro-osmotic behavior of ionic hybrid nanofluids in microchannels was theoretically investigated by Das et al. [40]. In a sinusoidal wavy heated tube with high zeta potential, Saleem et al. [41] examined the electric double layer (EDL)-induced flow and heat transfer of Casson fluid, emphasizing the important role the Debye–Huckel parameter plays in physiological fluid dynamics. EDL-driven biofluid transport across a microchannel was theoretically analyzed by Noreen et al. [42], who found that the fluid pressure gradient decreased as the electro-osmotic parameter values increased. Similarly, employing a non-Newtonian blood model, Akhtar et al. [43] offered hemodynamic insights into electro-osmotic flow in sick arteries, showing that electro-osmotic parameters can be tuned to influence blood flow successfully. Asghar et al. [44] discovered that in non-uniform diverging microchannels, the pressure gradient rises as the Helmholtz–Smoluchowski velocity and Debye–Hückel parameter increase. Additionally, EDL-induced streaming of ionized hybrid nano-blood through an eccentrically charged artery section with an endoscopic tube was assessed by Das et al. [45], who demonstrated improved fluid mobility under higher electro-osmotic conditions. In their investigation of fractional second-grade hybrid nanofluid flow through a stenosed and aneurysmal endoscopic arterial canal, Mehmood et al. [46] concluded that blood velocity increases as magnetic field intensity, electro-osmotic forces, and Helmholtz–Smoluchowski velocity increase.

Yuan et al. [47] investigated the evolution of electrokinetic vortices in transitioning microchannels, demonstrating the shift from two-dimensional confinement to three-dimensional chaotic mixing and its significance in enhancing microfluidic transport phenomena.

In arterial structures, the electrical double layer typically exhibits greater thickness in relation to the characteristics of the vessel wall and is modulated by intricate biological surfaces, including the endothelial lining, glycocalyx, ionic concentrations, and constituents of blood. The electrical double layer may exhibit spatial variability as a consequence of the elastic properties of the vessel and prevailing physiological conditions. Blood circulation within arterial systems is predominantly dictated by the forces arising from pressure gradients, viscous interactions, magnetic effects (as observed in MHD studies), pulsatile dynamics, and biological interactions. Although electro-osmotic effects are generally regarded as secondary, they may gain significance in the context of smaller vessels or pathological arterial conditions. In the context of arterial systems, the flow is predominantly governed by the pressure produced from cardiac contractions, while electro-osmotic phenomena play a supportive or regulatory role. The interplay between electro-osmotic and hemodynamic forces significantly influences velocity distributions, wall shear stress, and various transport phenomena. In general, arterial flow fields are curved, pulsatile, complicated, and may be disrupted by aneurysms, stenosis, or interactions with nanoparticles. Curvature and physiological geometry can cause asymmetric velocity profiles and secondary flows.

Using non-Newtonian fluid models, recent research has thoroughly examined blood flow dynamics, especially with regard to targeted medicine administration. However, we did not find any studies that focused on how Joule heating, radial magnetic fields, electro-osmosis, and nanoparticle size interact to affect flow through a curved stenosed artery with an aneurysm. In this work, we examine the flow of magnetite nanoparticles embedded in blood via an aneurysm in an irregularly stenosed artery. Electro-osmosis and a uniform radial magnetic field also affect the flow. We use a hematocrit-dependent viscosity model to accurately simulate the non-Newtonian behavior of blood. The governing equations are made simpler by using a curvilinear coordinate system and moderate stenosis assumptions. The Crank–Nicolson method is then used to discretize these equations, and they are subsequently solved numerically with appropriate boundary conditions. The current study’s novelty comprises the following:

- Investigation of the effect of a *Fe*_3_*O*_4_-based nanofluid on hemodynamics in stenosed and aneurysmal arterial segments.
- Analysis of how resistance and flow dynamics are affected by hematocrit-dependent blood viscosity.
- Evaluation of the Casson fluid parameter’s function in modulating non-Newtonian blood flow properties.
- Simulation of the impact of the nanoparticle’s size, volume fraction, and particle mass parameter on the hemodynamic of the blood flow.
- Incorporation of Corcione’s model of thermal conductivity and viscosity of the nanofluid.

## 2. Model Formulation

**Figure 1:**
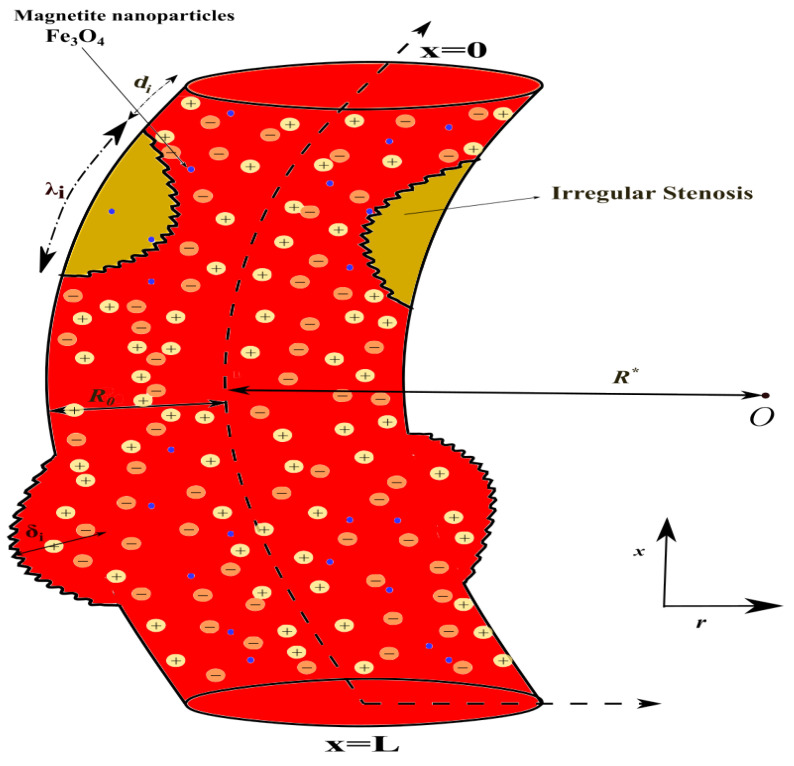
An illustration of a diseased artery in geometric form.

This study examines the fully developed, axisymmetric, and transient laminar motion of an incompressible Casson fluid that simulates blood flow via a finite-length curved artery, as shown in Figure 1. At the same time, segments with aneurysms and irregular stenosis have been taken into consideration. The flow behavior via the curved artery is investigated using (r,x), which are two-dimensional orthogonal curvilinear coordinates where *x*-axial direction, *r*-radial direction, u(r,x,t) = axial velocity component, v(r,x,t)) = radial velocity component. The flow direction (*x*-axis) receives a normal application of a uniform magnetic field (*B*). The current model is designed as an idealized conceptual structure for studying coupled transport phenomena under externally applied fields. Instead of capturing the precise field produced by a particular device shape, the selected magnetic field distribution is an idealistic approximation designed to simulate the impact of a non-uniform externally supplied magnetic field in a diseased artery. This model offers an idealized representation of curved artery flow. In the current investigation, the transport dynamics of *Fe*_3_*O*_4_ nanoparticles are conceptualized under the premises of dilute concentration, low Reynolds number, and diminutive particle size, wherein magnetic and viscous drag forces are posited as the pre-eminent influences. Consequently, more intricate hydrodynamic phenomena such as virtual mass, Basset history, and lift forces are omitted to create a simplified theoretical framework for analyzing the combined effects of electro-osmosis, magnetic field, curvature, and stenosis on the dynamics of blood flow.

The current study has the following constraints:

- Induced magnetic and electric fields are disregarded since the magnetic Reynolds number has been considered to be extremely low.
- Blood velocity and temperature will not alter along the axial direction (x-axis) because the flow is taken as fully developed.
- Order-analysis ($\epsilon =\left(\frac{R_{0}}{\lambda_{i}}\right)=O\left(1\right)$) and mild stenotic condition ($\delta =(\frac{\delta^{*}}{R_{0}})<<1$).
- Inertia of nanoparticles is neglected.
- It is anticipated that significant drag contact causes particle velocities to quickly equilibrate with the fluid velocity.
- Before using the asymptotic approximation $v_{p}\approx v_{f}$, the retained coupling term comes from the two-phase formulation.
- The constant zeta potential and Debye–Hückel approximation are adopted for numerical simplification purposes and might have limited utility under complicated physiological conditions.
- Electro-osmotic effects in curved arteries are comparatively less stronger than in microchannels.
- Fully 3-D Dean vortex structures are ignored by the axisymmetric assumption and lateral nanoparticle movement caused by secondary flow is not explicitly captured.

### 2.1. Diseased Segment Geometry

The following is a mathematical definition of the diseased segments:

(1) $$R\left(x\right)=\left\{\begin{array}{l} R_{0}-2\delta_{i}^{*}\left[\cos\left(\frac{2\pi }{\lambda_{i}}\left(\frac{x-d_{i}}{2}-\frac{\lambda_{i}}{4}\right)\right)-\frac{7}{100}\cos\left(\frac{32\pi }{\lambda_{i}}\left(x-d_{i}-\frac{\lambda_{i}}{2}\right)\right)\right],\;\;\;\;d_{i}\leq x\leq d_{i}+\lambda_{i},\;\;\;i=1,2\\ \;R_{0},\;\mathrm{otherwise}, \end{array}\right.$$

where −R(x) defines the lower arterial wall and R(x) indicates the upper arterial wall’s shape. The arterial segment is *L* in length, with $\lambda_{i}$ denoting the length of the $i^{\mathrm{th}}$ diseased region and $d_{i}$ indicating the axial position of the $i^{\mathrm{th}}$ aberrant section measured from the origin. $\delta_{i}^{*}$ represents the crucial height of the $i^{\mathrm{th}}$ diseased section at two distinct sites, as indicated by: $x=d_{1}+\frac{\lambda_{1}}{2},\;\mathrm{and}\;x=d_{2}+\frac{\lambda_{2}}{2},$ a stenotic segment (narrowing) is represented by $\delta_{i}^{*}>0$, while an aneurysmal segment (dilation) is indicated by $\delta_{i}^{*}<0$. The second segment is thought to have an aneurysm, while the first diseased section is thought to be stenotic in the current computational research.

### 2.2. Mathematical Formulation

#### 2.2.1. Electrohydrodynamics (EHD)

Blood has properties of an electrically conductive fluid because it contains a variety of ionic species in addition to components including hemoglobin, plasma, and leukocytes (white blood cells). As a result, this study examines how the dynamics of blood flow are affected by an externally supplied magnetic and electric field. While the electric field is written as $\left(0,0,E_{0}\right)$, where $B_{0}$ and $E_{0}$ are constants and R* is a reference radial coordinate, the magnetic field is defined as $B=(\frac{R^{*}B_{0}}{R^{*}+r},0,0)$. The electromagnetic body force acting on the fluid can be calculated using the Lorentz force law as follows:

(2) $$J\times B=\left(0,\sigma_{nf}\left(\frac{R^{*}}{r+R^{*}}\right)B_{0}E_{0},-\sigma_{nf}{\left(\frac{R^{*}B_{0}}{r+R^{*}}\right)}^{2}v\right),$$

(3) $$\frac{J.J}{\sigma_{nf}}=\sigma_{nf}{\left(\frac{R^{*}B_{0}}{r+R^{*}}\right)}^{2}{\left(v\right)}^{2}+\sigma_{nf}E_{0}^{2},$$

where $\sigma_{nf}$ and *J* stand for electric conductivity and current density respectively. When an electrolyte solution (blood) and solid conduct (arterial walls) come into contact a phenomenon known as electro-osmotic occurs. Because of the difference in concentration of ions, an electrical double layer (EDL) (a thin layer) is formed close to the surface. The Poisson–Boltzmann equation yields the following as the electro-osmotic potential function:

(4) $$\nabla^{2}\phi_{0}=-\frac{\rho_{e}}{\Upsilon },$$

where, $\phi_{0}$ is the electro-osmotic function,Υ is the dielectric constant, and $\rho_{e}$ is given by:

(5) $$\rho_{e}=\left(n^{+}-n^{-}\right)e_{0}z_{0}.$$

Boltzmann’s distribution number density of cations and anions is defined as follows:

(6) $$n^{\pm }=n_{0}\exp\left(\pm \frac{e_{0}z_{0}\phi_{0}}{k_{B}T_{avg}}\right),$$

where $e_{0}$ is electric constant, $z_{0}$ is charge balance, and $k_{B}$ is the Boltzmann constant.

Using the Debye-Huckel linearization, the Poisson equation takes the form:
  (7) $$\left(\frac{\partial^{2}}{\partial r^{2}}+\frac{1}{R^{*}+r}\frac{\partial }{\partial r}+{\left(\frac{R^{*}}{R^{*}+r}\right)}^{2}\frac{\partial^{2}}{\partial x^{2}}\right)\phi_{0}=\frac{\phi_{0}}{q_{m}^{2}},$$
  where $q_{m}=\frac{1}{e_{0}z_{0}}\sqrt{\frac{k_{B}\Upsilon T_{avg}}{2n_{0}}}$.

#### 2.2.2. Viscosity and Thermal Conductivity Model

Nanoparticle volume fraction and the size of the particles play a vital role in flow dynamics. As a result, in our study, the effect of particle size on the thermal conductivity and viscosity of nanofluids is taken into account. Using Corcione’s model, the thermal conductivity and viscosity of the nanofluid is given as:

(8) $$\frac{k_{nf}}{k_{f}}=1+4.4Re_{p}^{0.4}Pr^{0.66}{\left(\frac{T_{f}}{T_{fr}}\right)}^{10}{\left(\frac{k_{p}}{k_{f}}\right)}^{0.03}\phi_{1}^{0.66}$$

(9) $$\frac{\mu_{nf}}{\mu_{f}}=\frac{1}{1-34.87{\left(\frac{dp}{df}\right)}^{-0.3}\phi_{1}^{1.03}}$$

where, $df=0.1\frac{6M_{a}}{N_{a}\pi \rho_{f}}$ and $Re_{p}=\frac{2\rho_{f}{\bar{k}}_{b}T_{f}}{\pi dp{\left(\mu_{f}\right)}^{2}}$ = Nanoparticle Reynolds number, $k_{nf}$ = Thermal conductivity of nanofluid, $\mu_{nf}=$ Viscosity of nanofluid, $k_{p}=$ Nanoparticle’s thermal conductivity, $\mu_{f}=$ Blood viscosity, $k_{f}=$ Blood’s thermal conductivity, $T_{f}=$ Temperature of the blood, df= Diameter of the blood cells, $\phi_{1}=$ Volume fraction of nanoparticles. $T_{fr}=$ Blood freezing temperature, dp= Nanoparticles diameter, $M_{a}=$ Molecular weight of blood, $N_{a}=$ Avogadro number, ${\bar{k}}_{b}=1.38064\times 10^{-23}$ (Boltzmann constant).

Mathematical formulation of hematocrit-dependent viscosity is illustrated as follows:

(10) $$\mu_{f}=\mu_{0}\left[1+\beta_{1}h\left(r\right)\right],$$

where $h\left(r\right)=h_{m}\left[1-{\left(\frac{r}{R_{0}}\right)}^{m_{0}}\right]$, $h_{m}$ = maximum hematocrit at the center of the artery.

$\beta_{1}=2.5$, and $m_{0}$ = exact shape of velocity profile, $m_{0}\geq 2$.

According to the Brinkman model, the remaining thermal characteristics of nanofluid are expressed as:

(11) $$\rho_{nf}=\left(1-\phi_{1}\right)\rho_{f}+\phi_{1}\rho_{s_{1}},$$

(12) $${\left(\rho C_{p}\right)}_{nf}=\left(1-\phi_{1}\right){\left(\rho C_{p}\right)}_{f}+\phi_{1}{\left(\rho C_{p}\right)}_{s_{1}},$$

(13) $$\eta_{nf}=\left(1-\phi_{1}\right)\eta_{f}+\phi_{1}\eta_{s_{1}},$$

(14) $$\sigma_{nf}=\sigma_{f}\left[\frac{\sigma_{s_{1}}\left(1+\left(m-1\right)\phi_{1}\right)+\left(m-1\right)\sigma_{f}\left(1-\phi_{1}\right)}{\sigma_{s_{1}}\left(1-\phi_{1}\right)+\sigma_{f}\left(\left(m-1\right)+\phi_{1}\right)}\right],$$

where $\rho_{nf}=$ Density of nanofluid, $\sigma_{nf}=$ Electrical conductivity of nanofluid, ${\left(\rho C_{p}\right)}_{nf}=$ Heat capacity of nanofluid, $\eta_{nf}=$ Thermal expansion coefficient of nanofluid, $\rho_{f}=$ Density of blood, $\sigma_{f}=$ Electrical conductivity of blood, ${\left(\rho C_{p}\right)}_{f}=$ Heat capacity of blood, $\eta_{f}=$ Thermal expansion coefficient of blood, $\rho_{s_{1}}=$ Density of nanoparticle, $\sigma_{s_{1}}=$ Electrical conductivity of nanoparticle, ${\left(\rho C_{p}\right)}_{s_{1}}=$ Heat capacity of nanoparticle, $\eta_{s_{1}}=$ Thermal expansion coefficient of nanoparticle.

#### 2.2.3. Governing Equations

**Figure 2:**
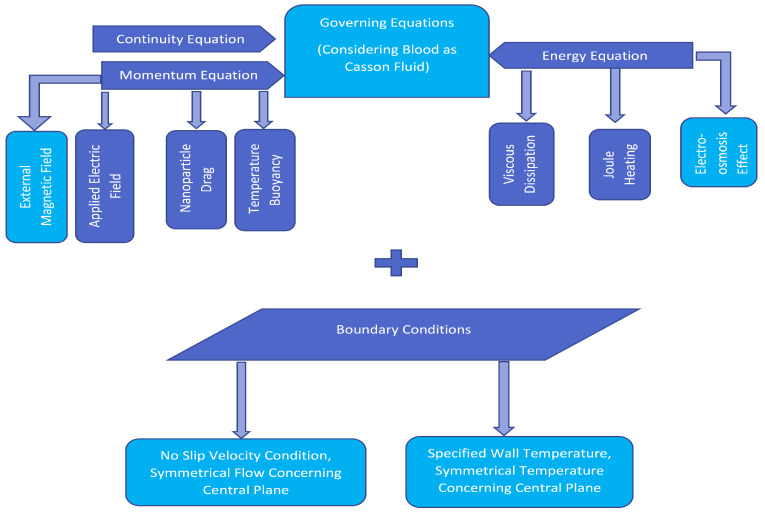
Flow chart of Mathematical model.

The study of curved arterial blood flow with stenosis and aneurysm is quite different from normal arterial flow because stresses and resistances are higher due to constrictions. The mathematical model used in the present study is illustrated in Figure 2. In low-shear or microvascular situations, when Newtonian models fall short in capturing significant physiological consequences, blood can be taken out as a Casson fluid to more accurately depict its true rheological behavior. The Casson fluid model’s rheological equation of state for an incompressible flow is as follows:

(15) $$\tau_{ij}^{*}=\left\{\begin{array}{c} \begin{array}{cc} 2\left(\mu_{f}^{*}+\frac{p_{y}^{*}}{\sqrt{2\pi^{*}}}\right)e_{ij}^{*},\; & \pi^{*}>\pi_{c}^{*}\\ 2\left(\mu_{f}^{*}+\frac{p_{y}^{*}}{\sqrt{2\pi_{c}^{*}}}\right)e_{ij}^{*},\; & \pi^{*}\leq \pi_{c}^{*} \end{array} \end{array}\right.$$

where $\pi^{*}=e_{ij}^{*}.e_{ij}^{*}$ is the product of deformation rate with itself, $\pi_{c}^{*}$ is a critical value based on the non-Newtonian model, $\mu_{f}^{*}$ is the plastic dynamic viscosity of the non-Newtonian fluid, and $p_{y}^{*}$ is the yield stress of the fluid. When $\pi^{*}\leq \pi_{c}^{*}$, the above equation can be expressed as:

(16) $$\tau_{ij}^{*}=2\mu_{f}^{*}\left(1+\frac{1}{\beta }\right)e_{ij}^{*}$$

where $\beta =\frac{\mu_{f}^{*}\sqrt{2\pi_{c}^{*}}}{p_{y}^{*}}$ is the Casson fluid parameter.

Under the above assumptions and invoking the Boussinesq approximation, the equations governing the flow are represented as: The continuity, momentum, energy, and concentration equations are represented as follows:

**Continuity Equation**

(17) $$\frac{\partial u}{\partial r}+\frac{u}{R^{*}+r}+\frac{R^{*}}{R^{*}+r}\frac{\partial v}{\partial x}=0.$$

**Momentum Equationsr-direction**

(18) $$\begin{array}{c} \rho_{nf}[\frac{\partial u}{\partial t}+u\frac{\partial u}{\partial r}+\frac{R^{*}v}{R^{*}+r}\frac{\partial v}{\partial x}-\frac{v^{2}}{R^{*}+r}]=-\frac{\partial P}{\partial r}+\left(1+\frac{1}{\beta }\right)\mu_{nf}[\frac{\partial^{2}u}{\partial r^{2}}+\frac{1}{R^{*}+r}\frac{\partial u}{\partial r}+{\left(\frac{R^{*}}{R^{*}+r}\right)}^{2}\frac{\partial^{2}u}{\partial x^{2}}\\ \;\;\;\;\;\;\;\;\;\;\;\;\;-\frac{u}{{\left(R^{*}+r\right)}^{2}}-\frac{2R^{*}}{{\left(R^{*}+r\right)}^{2}}\frac{\partial v}{\partial x}]+\left(\frac{4}{3}\frac{\partial u}{\partial r}-\frac{2}{3}\left(\frac{R^{*}}{R^{*}+r}\frac{\partial v}{\partial x}+\frac{u}{R^{*}+r}\right)\right)\left(1+\frac{1}{\beta }\right)\frac{\partial \mu_{nf}}{\partial r}+K_{s}N\left(v_{pr}^{*}-u\right), \end{array}$$

**x-direction**

(19) $$\begin{array}{c} \rho_{nf}[\frac{\partial v}{\partial t}+u\frac{\partial v}{\partial r}+\frac{R^{*}v}{R^{*}+r}\frac{\partial v}{\partial x}+\frac{uv}{R^{*}+r}]=-\frac{R^{*}}{R^{*}+r}\frac{\partial P}{\partial x}+\left(1+\frac{1}{\beta }\right)\mu_{nf}[\frac{\partial^{2}v}{\partial r^{2}}+\frac{1}{R^{*}+r}\frac{\partial v}{\partial r}\\ \;\;\;\;\;\;\;\;\;\;\;\;\;+{\left(\frac{R^{*}}{R^{*}+r}\right)}^{2}\frac{\partial^{2}v}{\partial x^{2}}-\frac{v}{{\left(R^{*}+r\right)}^{2}}+\frac{2R^{*}}{{\left(R^{*}+r\right)}^{2}}\frac{\partial u}{\partial x}]+\left(\frac{R^{*}}{R^{*}+r}\frac{\partial u}{\partial x}+\frac{\partial v}{\partial r}-\frac{v}{R^{*}+r}\right)\left(1+\frac{1}{\beta }\right)\frac{\partial \mu_{nf}}{\partial r}\\ \;\;\;\;\;\;\;\;\;\;\;\;\;+g{\left(\rho \eta \right)}_{nf}\left(T-T_{w}\right)+\rho_{e}E_{0}-\sigma_{nf}B_{0}^{2}v{\left(\frac{R^{*}}{R^{*}+r}\right)}^{2}+K_{s}N\left(v_{px}^{*}-v\right). \end{array}$$

**Energy Equation**

(20) $${\left(\rho C_{p}\right)}_{nf}\left[\frac{\partial T}{\partial t}+u\frac{\partial T}{\partial r}+\frac{R^{*}v}{R^{*}+r}\frac{\partial T}{\partial x}\right]=k_{nf}\left[\frac{\partial^{2}T}{\partial r^{2}}+\frac{1}{R^{*}+r}\frac{\partial T}{\partial r}+{\left(\frac{R^{*}}{R^{*}+r}\right)}^{2}\frac{\partial^{2}T}{\partial x^{2}}\right]+F_{vd}+\sigma_{nf}{\left(\frac{R^{*}B_{0}}{R^{*}+r}\right)}^{2}\left(v^{2}\right)+\sigma_{nf}E_{0}^{2}.$$

The Equation for the axial pressure gradient is defined as follows $-\frac{\partial \bar{P}}{\partial \bar{x}}=A_{0}+A_{1}\cos\left(2\pi \omega_{p}t\right),\;\;t>0,$ where $A_{0}=$ Mean pressure gradient, $\omega_{p}=2\pi f_{p}$, $A_{1}=$ Amplitude of the pulsatile component that maintains systolic and diastolic pressures, $F_{vd}=$ Dimensional form of viscous dissipation.

(21) $$\begin{array}{cc} F_{vd} & =\mu_{nf}\left(1+\frac{1}{\beta }\right)\left[2{\left(e_{rr}\right)}^{2}+2{\left(e_{xx}\right)}^{2}+{\left(e_{rx}\right)}^{2}\right]\\ \; & =\mu_{nf}\left(1+\frac{1}{\beta }\right)\left[2{\left(\frac{\partial u}{\partial r}\right)}^{2}+2{\left(\frac{R^{*}}{\left(R^{*}+r\right)}\frac{\partial v}{\partial x}+\frac{u}{\left(R^{*}+r\right)}\right)}^{2}+{\left(\frac{\partial v}{\partial r}-\frac{v}{\left(R^{*}+r\right)}+\frac{R^{*}}{\left(R^{*}+r\right)}\frac{\partial u}{\partial x}\right)}^{2}\right] \end{array}$$

Initial and boundary conditions associated with the flow are:

(22) $$\left\{\begin{array}{c} v=T=0\;\mathrm{at}\;\;\;t=0,\\ v=0,\;\;T=T_{w}\;\mathrm{at}\;\;\;r=R\;\;\;and\;\;\;r=-R. \end{array}\right.$$

**Electro-osmotic Equation**

(23) $$\nabla^{2}\phi_{0}=-\frac{\phi_{0}}{q_{m}^{2}}$$

where $\nabla^{2}\equiv \frac{\partial^{2}}{\partial r^{2}}+\frac{1}{R^{*}+r}\frac{\partial }{\partial r}+{\left(\frac{R^{*}}{R^{*}+r}\right)}^{2}\frac{\partial^{2}}{\partial x^{2}}$.

Boundary conditions for potential functions are defined as:

(24) $$\phi_{0}=\zeta_{w}\;\mathrm{at}\;\;\;r=R\;\;\;and\;\;\;r=-R.$$

where $\zeta_{w}$ represents potential functions defined on the arterial wall.

### 2.3. Motion of Nanoparticle

**Figure 3:**
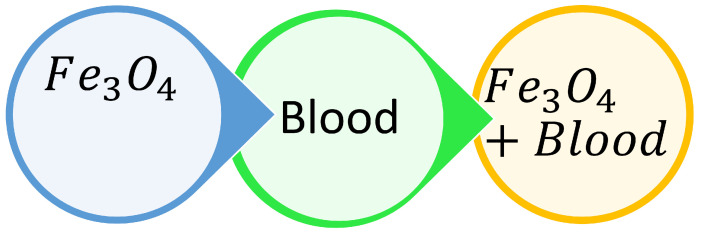
Combining magnetite nanoparticles in blood.

The labeled schematic diagram of combining magnetite nanoparticles in blood is presented in Figure 3. The motion of a spherical-shaped magnetic nanoparticle is considered in the flow of blood under the influence of applied magnetic field:

(25) $$m\frac{\partial v_{pr}^{*}}{\partial t}=F_{mr}+F_{fr}+F_{gr},$$

(26) $$m\frac{\partial v_{px}^{*}}{\partial t}=F_{mx}+F_{fx}+F_{gx}.$$

When the inertial term $m\frac{\partial v_{pr}^{*}}{\partial t}$ and $m\frac{\partial v_{px}^{*}}{\partial t}$ is ignored due to thenegligible mass of the nanoparticles, the particle motion satisfies the following simple force balance $F_{mr}+F_{fr}+F_{gr}=0$ and $F_{mx}+F_{fx}+F_{gx}=0$.

Fluidic force exerted by a nanoparticle is obtained by using Stokes’ law for the viscous drag on the sphere as follows:

(27) $$F_{fr}=-6\pi \mu R_{hyd,p}f_{Dp}\left(v_{pr}^{*}-u\right),$$

(28) $$F_{fx}=-6\pi \mu R_{hyd,p}f_{Dp}\left(v_{px}^{*}-v\right).$$

In this study, we ignored gravity’s effects on submicron nanoparticles and focused instead on the magnetic and fluidic forces. When the force on the nanoparticle is fluidic, the above equations become

(29) $$m\frac{\partial v_{pr}^{*}}{\partial t}=K_{s}\left(u-v_{pr}^{*}\right),$$

(30) $$m\frac{\partial v_{px}^{*}}{\partial t}=K_{s}\left(v-v_{px}^{*}\right),$$

where $K_{s}=6\pi \mu R_{hyd,p}f_{Dp}$ is the Stoke’s constant.

## 3. Non-Dimensional Equations

After using the non-dimensional parameter from Table 1 and mild stenotic condition, i.e., $\delta (=\frac{\delta^{*}}{R_{0}})<<1$, $\epsilon \left(=\frac{R_{0}}{\lambda_{i}}\right)=O\left(1\right)$, Equations (17)–(23) will be reduced to:

(31) $$\frac{\partial \bar{P}}{\partial \bar{r}}=0,$$

(32) $$\begin{array}{cc} \frac{\rho_{nf}}{\rho_{f}}Re\frac{\partial \bar{v}}{\partial \bar{t}} & =-\frac{R_{c}}{R_{c}+\bar{r}}\frac{\partial \bar{P}}{\partial \bar{x}}++\frac{\mu_{nf}}{\mu_{0}}\left(1+\frac{1}{\beta }\right)\left[\frac{\partial^{2}\bar{v}}{\partial {\bar{r}}^{2}}+\frac{1}{R_{c}+\bar{r}}\frac{\partial \bar{v}}{\partial \bar{r}}-\frac{\bar{v}}{{\left(R_{c}+\bar{r}\right)}^{2}}\right]-(\frac{\partial \bar{v}}{\partial \bar{r}}+\\ & \frac{\bar{v}}{R_{c}+\bar{r}})\left(1+\frac{1}{\beta }\right)\frac{m_{0}\beta_{1}h_{m}{\bar{r}}^{m_{0}-1}}{1-34.87{\left(\frac{dp}{db}\right)}^{-0.3}\phi_{1}^{1.03}}+U_{h_{0}s}q_{e}^{2}\Phi -\frac{\sigma_{nf}}{\sigma_{f}}{\left(\frac{R_{c}}{R_{c}+\bar{r}}\right)}^{2}M^{2}\bar{v}+\frac{{\left(\rho \eta \right)}_{nf}}{{\left(\rho \eta \right)}_{f}}\left(Gr\theta \right)+R_{s}\left[{\bar{v}}_{px}-\bar{v}\right], \end{array}$$

(33) $$\frac{{\left(\rho C_{p}\right)}_{nf}}{{\left(\rho C_{p}\right)}_{f}}\frac{k_{f}}{k_{nf}}PrRe\frac{\partial \theta }{\partial \bar{t}}=\frac{\partial^{2}\theta }{\partial {\bar{r}}^{2}}+\frac{1}{R_{c}+\bar{r}}\frac{\partial \theta }{\partial \bar{r}}+\left(1+\frac{1}{\beta }\right)\left(\frac{k_{f}}{k_{nf}}\right)\left(\frac{\mu_{nf}}{\mu_{0}}\right)Br{\left[\frac{\partial \bar{v}}{\partial \bar{r}}-\frac{\bar{v}}{R_{c}+\bar{r}}\right]}^{2}+\frac{\sigma_{nf}}{\sigma_{f}}\frac{k_{f}}{k_{nf}}\left[{\left(\frac{R_{c}}{R_{c}+\bar{r}}\right)}^{2}BrM^{2}{\bar{v}}^{2}+S_{j}\right],$$

(34) $$\frac{\partial^{2}\Phi }{\partial {\bar{r}}^{2}}+\frac{1}{R_{c}+\bar{r}}\frac{\partial \Phi }{\partial \bar{r}}=q_{e}^{2}\Phi .$$

dimensionless form of the diseased segment is given by:

(35) $$R\left(\bar{x}\right)=\left\{\begin{array}{c} 1-2\delta_{i}\left[\cos\left(2\pi \left(\frac{\bar{x}-{\bar{d}}_{i}}{2}-\frac{1}{4}\right)\right)-\frac{7}{100}\cos\left(32\pi \left(\bar{x}-{\bar{d}}_{i}-\frac{1}{2}\right)\right)\right],\;\;\;\;{\bar{d}}_{i}\leq x\leq {\bar{d}}_{i}+1,\;\;\;i=1,2\\ \;\;1,\;\mathrm{otherwise}. \end{array}\right.$$

Using the non-dimensional variables, Equations (29) and (30) become:

(36) $$R_{s}\frac{\partial v_{pr}^{*}}{\partial \bar{t}}=\left(\bar{u}-v_{pr}^{*}\right),$$

(37) $$R_{s}\frac{\partial v_{px}^{*}}{\partial \bar{t}}=\left(\bar{v}-v_{px}^{*}\right),$$

where $R_{s}=\frac{mu_{0}}{K_{s}R_{0}^{2}}$ is the particle mass parameter.

The non-dimensionalized form of pressure gradient and body acceleration is given as: $\frac{\partial \bar{P}}{\partial \bar{x}}=B_{1}(1+e\cos\left(c_{1}\bar{t}\right)$ and $G\left(\bar{t}\right)=B_{2}\cos\left(c_{2}\bar{t}+\chi \right),$

where, $e=\frac{A_{1}}{A_{0}},\;c_{1}=\frac{2\pi R_{0}w_{p}}{u_{0}},\;B_{1}=\frac{A_{0}R_{0}^{2}}{\mu_{0}u_{0}},\;B_{2}=\frac{\bar{A_{0}}R_{0}^{2}}{\mu_{0}u_{0}},\;c_{2}=\frac{\bar{\omega_{2}}R_{0}}{u_{0}}.$

The related initial and boundary conditions that are subject to the flow are transformed in the non-dimensionalized form as follows:
  (38) $$\left\{\begin{array}{c} \bar{v}=\theta =0\;\mathrm{at}\;\;\;t=0,\\ \bar{v}=0,\;\theta =1\;\mathrm{at}\;\;\;r=R\;\;\;and\;\;\;r=-R. \end{array}\right.$$
  Mathematical expressions for hemodynamical factors such as resistive impedance, volumetric flow rate, wall shear stress (WSS), and heat transfer coefficient are as follows:
  (39) $$\tau_{w}={\left(\frac{\partial \bar{v}}{\partial \bar{r}}\right)}_{\bar{r}=R},$$
  (40) $$Q=\int_{-R}^{R}\bar{v}\bar{r}d\bar{r},$$
  (41) $$\lambda =\frac{L\left(\frac{\partial \bar{P}}{\partial \bar{x}}\right)}{Q},$$
  (42) $$Nu={\left(\frac{\partial \theta }{\partial \bar{r}}\right)}_{\bar{r}=R}.$$

## 4. Discretization of Governing Equations

**Figure 4:**
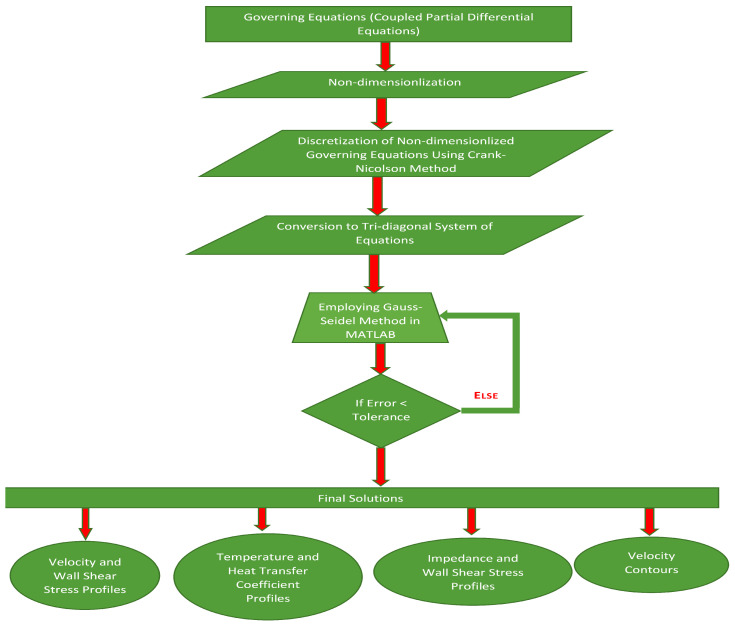
Flow chart representation of solution process.

The governing equations of the mathematical model are a collection of non-linear coupled partial differential equations. These equations cannot be handled accurately in all but a few extremely basic circumstances. Consequently, a number of distinct numerical techniques have been created to deal with these issues. Figure 4 presents the flow chart illustrating the solution methodology adopted in the present study. The Crank–Nicolson technique has been proposed as an implicit strategy by several researchers. With this method, the spatial derivative at $\left(t_{n-1/2},x_{j}\right)$ is replaced by averaging the upstream and downstream values at $t_{n-1}$ and $t_{n}$, respectively, using the finite difference grid. Likewise, substituting the central difference formula at $\left(t_{n-1/2},x_{j}\right)$ for the time derivative and employing thermophysical parameters and non-dimensional parameters reduced the governing equations to:

(43) $$\begin{array}{l} \left[\left(1-\phi_{1}\right)+\phi_{1}\frac{\rho_{s_{1}}}{\rho_{f}}\right]Re\left[\frac{{\bar{v}}_{i}^{k+1}-{\bar{v}}_{i}^{k}}{\Delta t}\right]=\frac{R_{c}}{R_{c}+\bar{r}\left(i\right)}D_{1}\left[1+ecos\left(c_{1}t^{k}\right)\right]\\ \;+\frac{\left[1+\beta_{1}h_{m}\left(1-{\left(\frac{\bar{r}\left(i\right)}{R_{0}}\right)}^{m_{0}}\right)\right]}{\left(1-34.87{\left(\frac{dp}{df}\right)}^{-0.3}\phi_{1}^{1.03}\right)}\left(1+\frac{1}{\beta }\right)[\frac{1}{2}\left(\frac{{\bar{v}}_{i+1}^{k+1}-2{\bar{v}}_{i}^{k+1}+{\bar{v}}_{i-1}^{k+1}}{{\left(\Delta x\right)}^{2}}+\frac{{\bar{v}}_{i+1}^{k}-2{\bar{v}}_{i}^{k}+{\bar{v}}_{i-1}^{k}}{{\left(\Delta x\right)}^{2}}\right)+\frac{1}{4(R_{c}+\bar{r}\left(i\right))}(\frac{{\bar{v}}_{i+1}^{k+1}-{\bar{v}}_{i-1}^{k+1}}{\Delta x}\\ \;+\frac{{\bar{v}}_{i+1}^{k}-{\bar{v}}_{i-1}^{k}}{\Delta x})-\frac{\left({\bar{v}}_{i}^{k}+{\bar{v}}_{i}^{k+1}\right)}{2{\left(R_{c}+\bar{r}\left(i\right)\right)}^{2}}]-\frac{m_{0}\beta_{1}h_{m}{\left(\bar{r}\left(i\right)\right)}^{m_{0}-1}}{\left(1-34.87{\left(\frac{dp}{df}\right)}^{-0.3}\phi_{1}^{1.03}\right)}\left(1+\frac{1}{\beta }\right)\left[\frac{\left({\bar{v}}_{i+1}^{k+1}-{\bar{v}}_{i-1}^{k+1}+{\bar{v}}_{i+1}^{k}-{\bar{v}}_{i-1}^{k}\right)}{4\Delta x}-\frac{\left({\bar{v}}_{i}^{k}+{\bar{v}}_{i}^{k+1}\right)}{2(R_{c}+\bar{r}\left(i\right))}\right]+\\ \;\left[\left(1-\phi_{1}\right)+\phi_{1}\frac{{\left(\rho \eta \right)}_{s1}}{{\left(\rho \eta \right)}_{f}}\right]\left(Gr\theta_{i}^{k}\right)+U_{h_{0}s}q_{e}^{2}\left(\Phi_{i}^{k}\right)-\frac{1}{2}\frac{\sigma_{nf}}{\sigma_{f}}M^{2}\left({\bar{v}}_{i}^{k}+{\bar{v}}_{i}^{k+1}\right){\left(\frac{R_{c}}{R_{c}+\bar{r}\left(i\right)}\right)}^{2}+\frac{R_{s}}{2}\left[\left({\bar{v}}_{{px}_{i}}^{k}+{\bar{v}}_{{px}_{i}}^{k+1}\right)-\left({\bar{v}}_{i}^{k}+{\bar{v}}_{i}^{k+1}\right)\right], \end{array}$$

(44) $$\begin{array}{c} \left[\left(1-\phi_{1}\right)+\phi_{1}\frac{{\left(\rho C_{p}\right)}_{s1}}{{\left(\rho C_{p}\right)}_{f}}\right]\left[\frac{\theta_{i}^{k+1}-\theta_{i}^{k}}{\Delta t}\right]=\frac{1}{RePr}\left(1+4.4Re_{p}^{0.4}Pr^{0.66}{\left(\frac{T_{f}}{T_{fr}}\right)}^{10}{\left(\frac{k_{p}}{k_{f}}\right)}^{0.03}\phi_{1}^{0.66}\right)[\left(\frac{\theta_{i+1}^{k+1}-2\theta_{i}^{k+1}+\theta_{i-1}^{k+1}}{2{\left(\Delta x\right)}^{2}}+\frac{\theta_{i+1}^{k}-2\theta_{i}^{k}+\theta_{i-1}^{k}}{2{\left(\Delta x\right)}^{2}}\right)\\ \;+\frac{1}{4(R_{c}+\bar{r}\left(i\right))}\left(\frac{\theta_{i+1}^{k+1}-\theta_{i-1}^{k+1}}{\Delta x}+\frac{\theta_{i+1}^{k}-\theta_{i-1}^{k}}{\Delta x}\right)]+\left(1+\frac{1}{\beta }\right)\left(\frac{\mu_{nf}}{\mu_{0}}\right)\left(\frac{Br}{RePr}\right){\left[\frac{\left({\bar{v}}_{i+1}^{k+1}-{\bar{v}}_{i-1}^{k+1}+{\bar{v}}_{i+1}^{k}-{\bar{v}}_{i-1}^{k}\right)}{4\Delta x}-\frac{\left({\bar{v}}_{i}^{k}+{\bar{v}}_{i}^{k+1}\right)}{2(R_{c}+\bar{r}\left(i\right))}\right]}^{2}\\ \;+\frac{1}{PrRe}\left\{\frac{\sigma_{nf}}{\sigma_{f}}\left[{\left(\frac{R_{c}}{R_{c}+\bar{r}\left(i\right)}\right)}^{2}BrM^{2}{\left({\bar{v}}_{i}^{2}\right)}^{k}+S_{j}\right]\right\}, \end{array}$$

(45) $$\frac{\Phi_{i+1}-2\Phi_{i}+\Phi_{i-1}}{h^{2}}+\frac{1}{R_{c}+\bar{r}\left(i\right)}\left\{\frac{\Phi_{i+1}-\Phi_{i-1}}{2h}\right\}=q_{e}^{2}\Phi_{i}.$$

Equation (43) can be written in a tri-diagonal system as:

(46) $$T_{i}^{k}{\bar{v}}_{i-1}^{k+1}+S_{i}^{k}{\bar{v}}_{i}^{k+1}+R_{i}^{k}{\bar{v}}_{i+1}^{k+1}=T_{i}^{\prime k}{\bar{v}}_{i-1}^{k}+S_{i}^{\prime k}{\bar{v}}_{i}^{k}+R_{i}^{\prime k}{\bar{v}}_{i+1}^{k}+F_{i}^{k},$$

where,

(47) $$T_{i}^{k}=\frac{\left[1+\beta_{1}h_{m}\left(1-{\left(\frac{\bar{r}\left(i\right)}{R_{0}}\right)}^{m_{0}}\right)\right]}{\left(1-34.87{\left(\frac{dp}{df}\right)}^{-0.3}\phi_{1}^{1.03}\right)}\left(1+\frac{1}{\beta }\right)\left(-\frac{\Delta t}{2{\left(\Delta x\right)}^{2}}+\frac{\Delta t}{4(R_{c}+\bar{r}\left(i\right))\left(\Delta x\right)}\right)-\frac{m_{0}\beta_{1}h_{m}{\left(\bar{r}\left(i\right)\right)}^{m_{0}-1}}{\left(1-34.87{\left(\frac{dp}{df}\right)}^{-0.3}\phi_{1}^{1.03}\right)}\left(1+\frac{1}{\beta }\right)\left(\frac{\Delta t}{4\Delta x}\right),$$

(48) $$\begin{array}{l} S_{i}^{k}=Re\left[\left(1-\phi_{1}\right)+\phi_{1}\frac{\rho_{s1}}{\rho_{f}}\right]-\frac{\left[1+\beta_{1}h_{m}\left(1-{\left(\frac{\bar{r}\left(i\right)}{R_{0}}\right)}^{m_{0}}\right)\right]}{\left(1-34.87{\left(\frac{dp}{df}\right)}^{-0.3}\phi_{1}^{1.03}\right)}\left(1+\frac{1}{\beta }\right)\left(-\frac{\Delta t}{{\left(\Delta x\right)}^{2}}-\frac{\Delta t}{2{\left(R_{c}+\bar{r}\left(i\right)\right)}^{2}}\right)\\ \;-\frac{m_{0}\beta_{1}h_{m}{\left(\bar{r}\left(i\right)\right)}^{m-1}}{\left(1-34.87{\left(\frac{dp}{df}\right)}^{-0.3}\phi_{1}^{1.03}\right)}\left(1+\frac{1}{\beta }\right)\left(\frac{\Delta t}{2(R_{c}+\bar{r}\left(i\right))}\right)+\frac{\Delta t}{2}\frac{\sigma_{nf}}{\sigma_{f}}M^{2}{\left(\frac{R_{c}}{R_{c}+\bar{r}\left(i\right)}\right)}^{2}+\frac{R_{s}\Delta t}{2}, \end{array}$$

(49) $$\begin{array}{c} R_{i}^{k}=-\frac{\left[1+\beta_{1}h_{m}\left(1-{\left(\frac{\bar{r}\left(i\right)}{R_{0}}\right)}^{m_{0}}\right)\right]}{\left(1-34.87{\left(\frac{dp}{df}\right)}^{-0.3}\phi_{1}^{1.03}\right)}\left(1+\frac{1}{\beta }\right)\left(\frac{\Delta t}{2{\left(\Delta x\right)}^{2}}+\frac{\Delta t}{4(R_{c}+\bar{r}\left(i\right))\Delta x}\right)+\frac{m_{0}\beta_{1}h_{m}{\left(\bar{r}\left(i\right)\right)}^{m_{0}-1}}{\left(1-34.87{\left(\frac{dp}{df}\right)}^{-0.3}\phi_{1}^{1.03}\right)}\left(1+\frac{1}{\beta }\right)\left(\frac{\Delta t}{4\Delta x}\right), \end{array}$$

(50) $$\begin{array}{c} T_{i}^{\prime k}=\frac{\left[1+\beta_{1}h_{m}\left(1-{\left(\frac{\bar{r}}{R_{0}}\right)}^{m_{0}}\right)\right]}{\left(1-34.87{\left(\frac{dp}{df}\right)}^{-0.3}\phi_{1}^{1.03}\right)}\left(1+\frac{1}{\beta }\right)\left(\frac{\Delta t}{2{\left(\Delta x\right)}^{2}}-\frac{\Delta t}{4(R_{c}+\bar{r}\left(i\right))\left(\Delta x\right)}\right)+\frac{m_{0}\beta_{1}h_{m}{\left(\bar{r}\left(i\right)\right)}^{m_{0}-1}}{\left(1-34.87{\left(\frac{dp}{df}\right)}^{-0.3}\phi_{1}^{1.03}\right)}\left(1+\frac{1}{\beta }\right)\left(\frac{\Delta t}{4\Delta x}\right), \end{array}$$

(51) $$\begin{array}{l} S_{i}^{\prime k}=Re\left[\left(1-\phi_{1}\right)+\phi_{1}\frac{\rho_{s1}}{\rho_{f}}\right]+\frac{\left[1+\beta_{1}h_{m}\left(1-{\left(\frac{\bar{r}\left(i\right)}{R_{0}}\right)}^{m_{0}}\right)\right]}{\left(1-34.87{\left(\frac{dp}{df}\right)}^{-0.3}\phi_{1}^{1.03}\right)}\left(1+\frac{1}{\beta }\right)\left(-\frac{\Delta t}{{\left(\Delta x\right)}^{2}}-\frac{\Delta t}{2{\left(R_{c}+\bar{r}\left(i\right)\right)}^{2}}\right)\\ \;+\frac{m_{0}\beta_{1}h_{m}{\left(\bar{r}\left(i\right)\right)}^{m_{0}-1}}{\left(1-34.87{\left(\frac{dp}{df}\right)}^{-0.3}\phi_{1}^{1.03}\right)}\left(1+\frac{1}{\beta }\right)\left(\frac{\Delta t}{2(R_{c}+\bar{r}\left(i\right))}\right)-\frac{\Delta t}{2}\frac{\sigma_{nf}}{\sigma_{f}}M^{2}{\left(\frac{R_{c}}{R_{c}+\bar{r}\left(i\right)}\right)}^{2}-\frac{R_{s}\Delta t}{2}, \end{array}$$

(52) $$\begin{array}{c} R_{i}^{\prime k}=\frac{\left[1+\beta_{1}h_{m}\left(1-{\left(\frac{\bar{r}}{R_{0}}\right)}^{m_{0}}\right)\right]}{\left(1-34.87{\left(\frac{dp}{df}\right)}^{-0.3}\phi_{1}^{1.03}\right)}\left(1+\frac{1}{\beta }\right)\left(\frac{\Delta t}{2{\left(\Delta x\right)}^{2}}+\frac{\Delta t}{4(R_{c}+\bar{r}\left(i\right))\Delta x}\right)-\frac{m_{0}\beta_{1}h_{m}{\left(\bar{r}\left(i\right)\right)}^{m_{0}-1}}{\left(1-34.87{\left(\frac{dp}{df}\right)}^{-0.3}\phi_{1}^{1.03}\right)}\left(1+\frac{1}{\beta }\right)\left(\frac{\Delta t}{4\Delta x}\right), \end{array}$$

(53) $$\begin{array}{c} F_{i}^{k}=\Delta t\frac{D_{1}R_{c}}{\left(R_{c}+\bar{r}\left(i\right)\right)}\left[1+e\cos\left(c_{1}t^{k}\right)\right]+\left(\Delta t\right)\left[\left(1-\phi_{1}\right)+\phi_{1}\frac{{\left(\rho \eta \right)}_{s_{1}}}{{\left(\rho \eta \right)}_{f}}\right]\left(Gr\theta_{i}^{k}\right)+\Delta tU_{h_{0}s}q_{e}^{2}\Phi_{i}^{k}+\frac{R_{s}\Delta t}{2}\left[\left({\bar{v}}_{{px}_{i}}^{k}+{\bar{v}}_{{px}_{i}}^{k+1}\right)\right]. \end{array}$$

Using Equation (44), the following tridiagonal system is obtained:

(54) $$A_{i}^{\prime k}\theta_{i-1}^{k+1}+B_{i}^{\prime k}\theta_{i}^{k+1}+C_{i}^{\prime k}\theta_{i+1}^{k+1}=A_{i}^{\prime \prime k}\theta_{i-1}^{k}+B_{i}^{\prime \prime k}\theta_{i}^{k}+C_{i}^{\prime \prime k}\theta_{i+1}^{k}+D_{i}^{\prime k},$$

where,

(55) $$A_{i}^{\prime k}=\frac{1}{RePr}(1+4.4Re_{p}^{0.4}Pr^{0.66}{\left(\frac{T_{f}}{T_{fr}}\right)}^{10}{\left(\frac{k_{p}}{k_{f}}\right)}^{0.03}\phi_{1}^{0.66})(-\frac{\Delta t}{2{\left(\Delta x\right)}^{2}}+\frac{1}{4(R_{c}+\bar{r}\left(i\right))}\frac{\Delta t}{\Delta x}),$$

(56) $$B_{i}^{\prime k}=[\left(1-\phi_{1}\right)+\phi_{1}\frac{{\left(\rho C_{p}\right)}_{s_{1}}}{{\left(\rho C_{p}\right)}_{f}}]+\frac{1}{RePr}(1+4.4Re_{p}^{0.4}Pr^{0.66}{\left(\frac{T_{f}}{T_{fr}}\right)}^{10}{\left(\frac{k_{p}}{k_{f}}\right)}^{0.03}\phi_{1}^{0.66})(\frac{\Delta t}{{\left(\Delta x\right)}^{2}}),$$

(57) $$C_{i}^{\prime k}=-\frac{1}{RePr}(1+4.4Re_{p}^{0.4}Pr^{0.66}{\left(\frac{T_{f}}{T_{fr}}\right)}^{10}{\left(\frac{k_{p}}{k_{f}}\right)}^{0.03}\phi_{1}^{0.66})(\frac{\Delta t}{2{\left(\Delta x\right)}^{2}}+\frac{1}{4(R_{c}+\bar{r}\left(i\right))}\frac{\Delta t}{\left(\Delta x\right)}),$$

(58) $$A_{i}^{\prime \prime k}=\frac{1}{RePr}(1+4.4Re_{p}^{0.4}Pr^{0.66}{\left(\frac{T_{f}}{T_{fr}}\right)}^{10}{\left(\frac{k_{p}}{k_{f}}\right)}^{0.03}\phi_{1}^{0.66})(\frac{\Delta t}{2{\left(\Delta x\right)}^{2}}-\frac{1}{4(R_{c}+\bar{r}\left(i\right))}\frac{\Delta t}{\Delta x}),$$

(59) $$B_{i}^{\prime \prime k}=[\left(1-\phi_{1}\right)+\phi_{1}\frac{{\left(\rho C_{p}\right)}_{s1}}{{\left(\rho C_{p}\right)}_{f}}]-\frac{1}{RePr}(1+4.4Re_{p}^{0.4}Pr^{0.66}{\left(\frac{T_{f}}{T_{fr}}\right)}^{10}{\left(\frac{k_{p}}{k_{f}}\right)}^{0.03}\phi_{1}^{0.66})\frac{\Delta t}{{\left(\Delta x\right)}^{2}},$$

(60) $$C_{i}^{\prime \prime k}=\frac{1}{RePr}(1+4.4Re_{p}^{0.4}Pr^{0.66}{\left(\frac{T_{f}}{T_{fr}}\right)}^{10}{\left(\frac{k_{p}}{k_{f}}\right)}^{0.03}\phi_{1}^{0.66})(\frac{\Delta t}{2{\left(\Delta x\right)}^{2}}+\frac{1}{4(R_{c}+\bar{r}\left(i\right))}\frac{\Delta t}{\Delta x}),$$

(61) $$D_{i}^{\prime k}=(1+\frac{1}{\beta })(\frac{\mu_{nf}}{\mu_{0}})(\frac{Br\Delta t}{RePr}){\left[\frac{\left({\bar{v}}_{i+1}^{k+1}-{\bar{v}}_{i-1}^{k+1}+{\bar{v}}_{i+1}^{k}-{\bar{v}}_{i-1}^{k}\right)}{4\Delta x}-\frac{\left({\bar{v}}_{i}^{k}+{\bar{v}}_{i}^{k+1}\right)}{2(R_{c}+\bar{r}\left(i\right))}\right]}^{2}+\frac{\Delta t}{PrRe}\left\{\frac{\sigma_{nf}}{\sigma_{f}}[{\left(\frac{R_{c}}{R_{c}+\bar{r}\left(i\right)}\right)}^{2}BrM^{2}{\left({\bar{v}}_{i}^{2}\right)}^{k}+S_{j})\right\}.$$

(N+1)×(M+1) grid points have been created from the flow region. Despite the second-order convergence of the Crank–Nicolson approach, we have taken into account the discretization of space and time with step sizes of x=0.001 and t=0.01. To verify the selected discretization parameters and guarantee the stability of the Crank–Nicholson scheme for the current nonlinear issue, mesh refinement and convergence checks were carried out. The appropriate meshing approach ensures convergence to the fifth order. To determine the velocity, temperature, and electro-osmotic distributions, MATLAB programming code is created. The electro-osmotic equation does not depend on changes in time. Thus, we created a function for it and included it in each temporal iteration.

## 5. Results and Graphical Analysis

**Figure 5:**
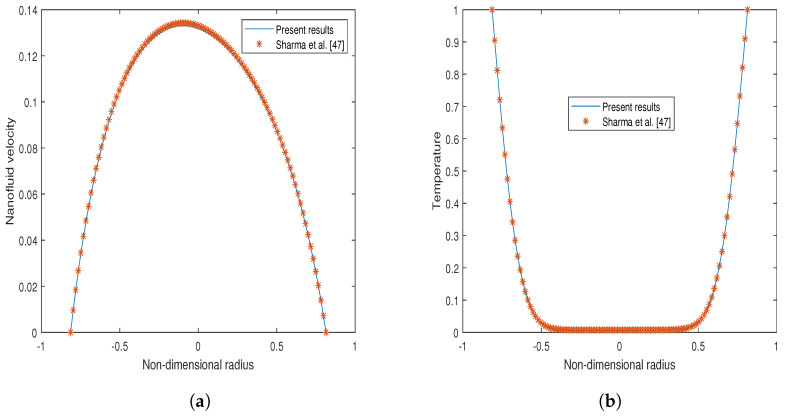
(**a**) Velocity validation at $\phi_{1}=0.02$, (**b**) Temperature validation at $\phi_{1}=0.02$ [47].

The published work by Sharma et al. [48] for the curved artery with stenosis and aneurysm, which is prevalent in both studies, completes the validation of our investigation. To authenticate the dimensionless velocity profile Figure 5a,b, for the $Al_{2}O_{3}$ nanoparticle, this validation has been carried out in a way that considers the radiation effect and ignores the impact of the Casson fluid parameter (β=0) and particle mass parameter ($R_{s}=0$). Here, Corcione’s model is only used to model the viscosity of the nanofluid. The Crank–Nicolson (C-N) is employed for the current work, with a tolerance of $10^{-6}$ for each iteration. For both temperature and velocity, it is found that the current study and the published work [48] correspond well, which confirms the validity of the current study. The thermophysical properties of blood and ($Fe_{3}O_{4}$) nanoparticles are listed in Table 2. The physical parameter values together with their ranges and corresponding sources are presented in Table 3. In addition, the default values of the physical parameters employed in the present computations are provided in Table 4.

### 5.1. Variation Concerning Casson Fluid Parameter

**Figure 6:**
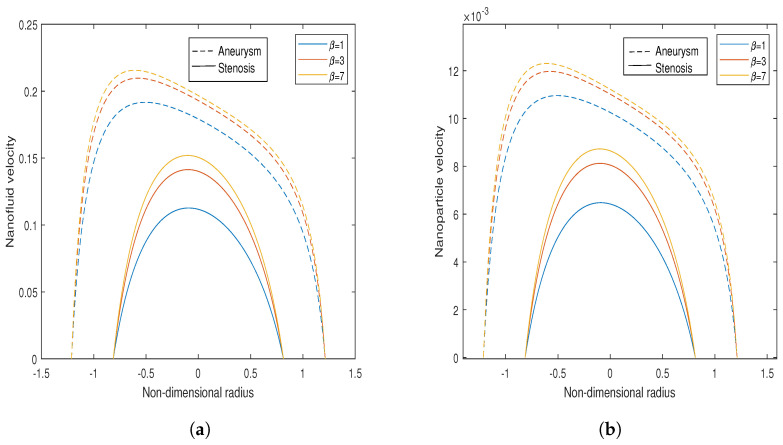
(**a**) Variation of nanofluid velocity w.r.t. Casson parameter. (**b**) Variation in nanoparticle velocity w.r.t. Casson parameter.

The effect of the Casson parameter β on the nanofluid velocity profile is shown in Figure 6a. As shear-thinning behavior in the nanofluid is encouraged, yield stress is reduced when the Casson parameter is increased. As a result, at greater shear rates, the effective viscosity decreases, lowering the flow resistance and raising the nanofluid’s velocity. The effect of the Casson parameter β on the nanoparticle’s velocity profile is represented in Figure 6b. A Eulerian continuum approximation is used to represent the nanoparticle phase, and no tracking of Lagrangian particles was done and the pictures show averaged nanoparticle velocity distributions rather than individual particle trajectories. The stated nanoparticle velocity reflects locally averaged particle motion derived from simplified force balancing equations. The following factors cause the velocity of $Fe_{3}O_{4}$ nanoparticles to increase with an increase in the Casson parameter, which leads to diminished yield stress and enhanced shear-thinning behavior. Also, magnetic and thermal improvements were reflected by ${Fe}_{3}O_{4}^{\prime }s$ improved thermal and magnetic characteristics, which is an additional factor in the favorable flow conditions of nanoparticles.

**Figure 7:**
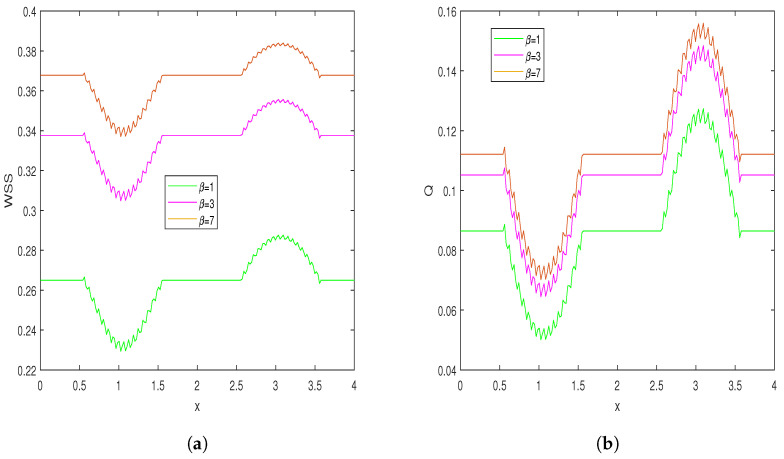
Variation of (**a**) wall shear stress and, (**b**) flow rate w.r.t. Casson fluid parameter.

Figure 7a,b demonstrate the variation in wall shear stress and flow rate respectively in the artery concerning the Casson fluid parameter. As the Casson fluid parameter rises, the fluid’s yield stress decreases and the fluid flows more freely. Consequently, there is less resistance in the fluid stream. The non-Newtonian behavior of blood is typically described in blood flow using the Casson fluid model. In contrast to Newtonian fluids, the Casson fluid model takes yield stress into account, which has an impact on the wall shear stress in a different way. In general, the wall shear stress tends to grow along with the Casson fluid parameter, which indicates increasing yield stress and viscosity. This is because increased boundary layer thickness and flow resistance brought about by increased viscosity and yield stress result in increased shear stresses that affect the vessel walls. Nevertheless, the relationship may be intricate and may change based on variables like flow rate, vessel geometry, and the particulars of the blood that is being simulated.

### 5.2. Variation Concerning Volume Fraction of Nanoparticles

**Figure 8:**
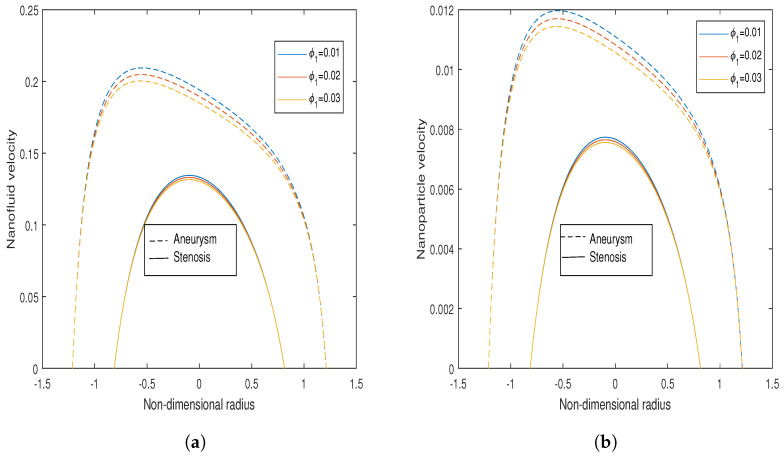
Variation in (**a**) nanofluid velocity and (**b**) nanoparticle velocity w.r.t. volume fraction of nanoparticles.

Figure 8a shows the decrease in nanofluid velocity with regard to the increment in the volume fraction of $Fe_{3}O_{4}$ nanoparticles. Increased viscosity and density, as well as improved particle interactions and shear stress within the fluid, are the main causes of the decrease in velocity of nanofluids with the increasing nanoparticle volume fraction. Figure 8b shows the decrease in nanoparticles velocity with regard to the increment in the volume fraction of $Fe_{3}O_{4}$ nanoparticles. In the bloodstream velocity of $Fe_{3}O_{4}$, the amount of nanoparticles decreases as the volume percentage of $Fe_{3}O_{4}$ increases. This is due to increased viscosity, improved magnetic and hydrodynamic interactions, greater frequency of particle–cell interactions, and change in the flow behavior of the suspended elements. These all contribute to the decrease in the velocity of nanoparticles in the circulation.

**Figure 9:**
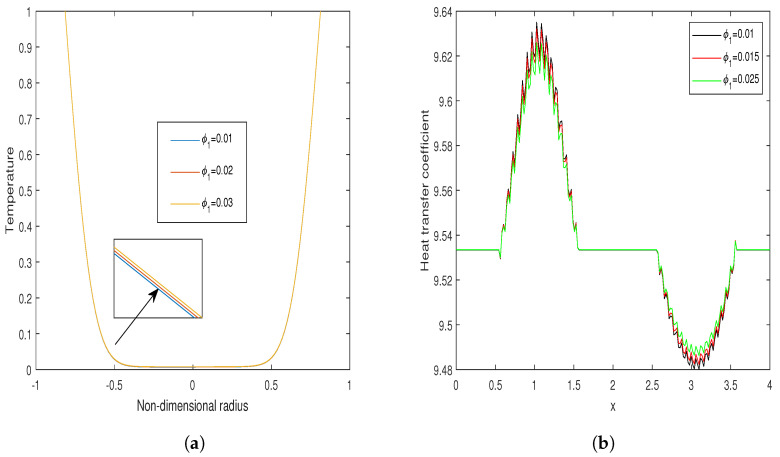
Variation of (**a**) temperature of nanofluid and (**b**) heat transfer coefficient w.r.t. volume fraction of nanoparticles.

Figure 9a demonstrates the variation of nanofluid temperature concerning different volume fractions of $Fe_{3}O_{4}$ nanoparticles. The nanofluid’s specific heat capacity and overall energy storage capacity can fluctuate, which can affect how the temperature dynamics during heating or cooling processes are affected. As the concentration of nanoparticles increases, the fluid’s convective heat transmission mechanisms may also be impacted. All these elements work together to improve a fluid’s thermal characteristics as the volume fraction of the nanoparticles in it rises. This results in more effective heat transmission and a rise in fluid temperature under the same heating circumstances. Figure 9b shows the change in heat transfer coefficient with variation in the volume fraction of $Fe_{3}O_{4}$ nanoparticles. The main causes of the decrease in the heat transfer coefficient with increasing nanoparticle volume fraction are changes in thermal diffusivity, thickening thermal boundary layer, particle agglomeration, increased viscosity, and specific heat capacity. These elements work together to provide more heat transfer resistance, resulting in a lesser heat transfer coefficient.

**Figure 10:**
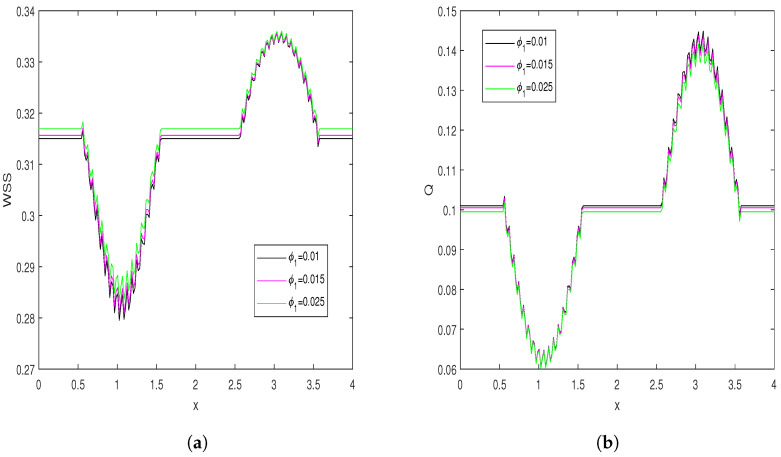
Variation in (**a**) wall shear stress and (**b**) flow rate w.r.t. $\phi_{1}$.

Figure 10a depicts the change in wall shear stress with the variation in the volume fraction of $Fe_{3}O_{4}$ nanoparticles. $Fe_{3}O_{4}$ nanoparticles may also affect blood vessel endothelial cells. Higher-volume percentages of nanoparticles may result in more noticeable interactions, which may harm or activate endothelial cells. The vessel walls may experience elevated shear stress as a result of these biological reactions. Figure 10b depicts the change in flow rate with the variation in the volume fraction of $Fe_{3}O_{4}$ nanoparticles. Nanoparticles have a stronger propensity to agglomerate and form bigger clusters or clumps at increasing volume fractions. When blood flow interacts with irregular surfaces or blockages caused by the aggregates, these aggregates may cause flow disruptions by decreasing the flow rate and increasing shear stress on the vessel walls.

### 5.3. Variation Concerning Particle Mass Parameter

**Figure 11:**
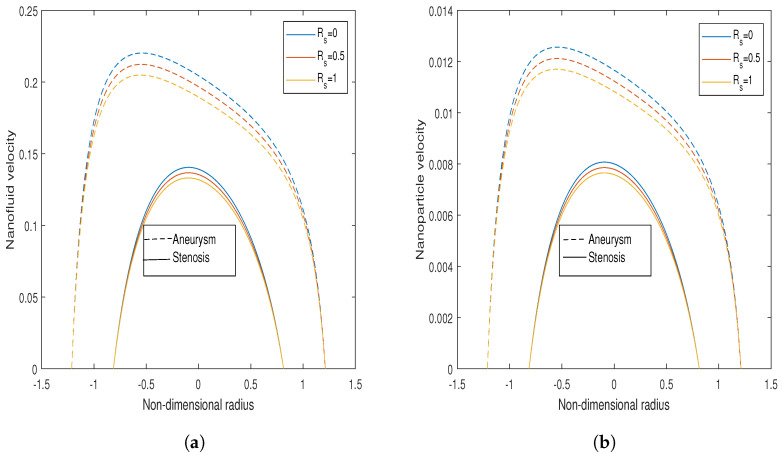
Variation of (**a**) nanofluid velocity, (**b**) nanoparticle velocity w.r.t. particle mass parameter.

Figure 11a shows the decrease in nanofluid velocity concerning the increment in particle mass parameter. Many variables, including increased viscosity, particle interactions, sedimentation, and thermal impacts, are responsible for the decrease in velocity in a nanofluid with an increase in the particle mass parameter. Figure 11b shows the decrease in nanoparticle velocity concerning the increment in particle mass parameter. Enhanced sedimentation, greater hydrodynamic interactions, a tendency towards agglomeration, decreased responsiveness to Brownian motion, and increased drag force are some of the factors that influence the decrease in nanoparticle velocity with an increase in the particle mass parameter. The combined effect of these factors causes the flow of heavier nanoparticles through the fluid to slow down.

**Figure 12:**
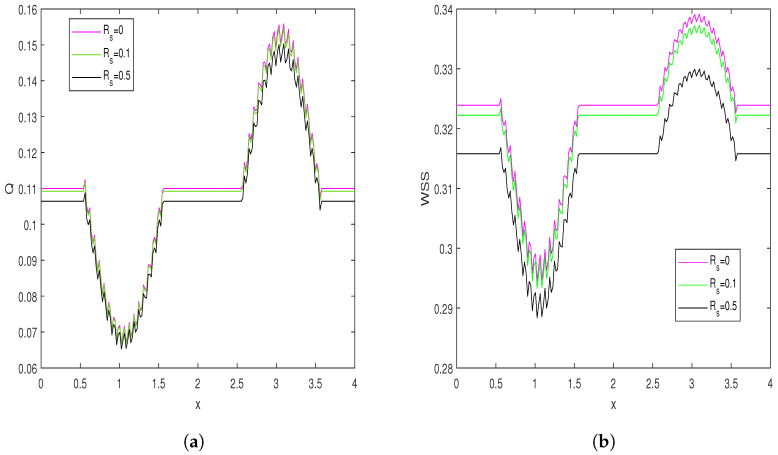
Variation in (**a**) flow rate and (**b**) wall shear stress w.r.t. $R_{s}$.

Figure 12a shows the change in blood flow rate with variation in particle mass parameter. The decrease in flow rate with the increasing particle mass parameter of $Fe_{3}O_{4}$ nanoparticles is mainly caused by higher effective viscosity, improved particle–particle interactions, stronger drag forces, as well as complex thermal and hydrodynamic actions. These elements work together to create more flow resistance, resulting in a slower flow rate.

Figure 12b shows the variation in arterial wall shear stress with a change in particle mass parameter. Greater aggregation and sedimentation of nanoparticles in the fluid can be caused by higher particle mass characteristics, such as increased particle density. This may have an impact on the nanoparticle distribution close to the wall, which may lead to a decrease in the WSS. Also, the fluid’s viscosity may change due to the presence of nanoparticles, which may have an impact on the WSS. Increased viscosity from improved particle–particle interactions may result from higher particle mass parameters, which may have an effect on the WSS distribution.

### 5.4. Variation Concerning Diameter of Nanoparticle

**Figure 13:**
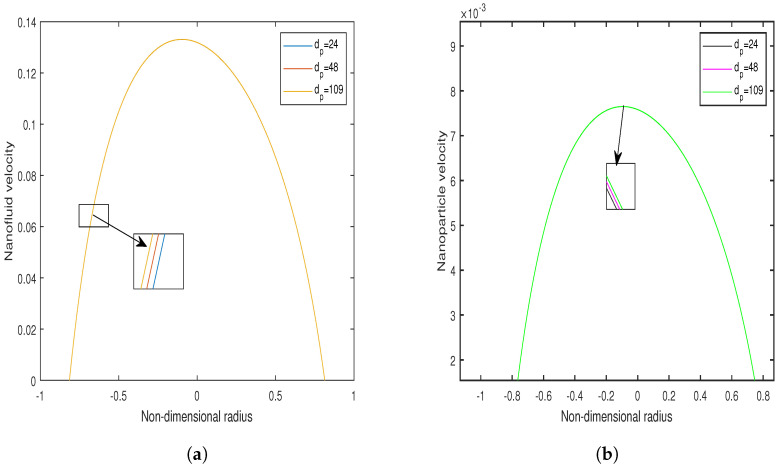
Variation of (**a**) nanofluid velocity, and (**b**) nanoparticle velocity w.r.t. diameter of nanoparticles.

Figure 13a shows the increase in nanofluid velocity concerning the increment in the diameter of the nanoparticle. Nanoparticles increase the thermal conductivity of the base fluid. With larger nanoparticles, this improvement could be more noticeable. Higher thermal conductivity can result in improved heat transmission and, thus, enhance fluid flow. Also, larger nanoparticles may have more robust contact with the base fluid. This can result in a more effective “push” from the fluid onto the nanoparticles, increasing the total velocity of the nanofluid. Figure 13b shows the increase in nanoparticle velocity with regard to the increment in the diameter of the nanoparticle. Larger nanoparticles might experience lower drag force per unit mass than smaller nanoparticles. Drag force is the resistance an object encounters while moving through a fluid. The surface-area-to-volume ratio decreases with increasing diameter. This means that larger nanoparticles have less surface area than their volume, resulting in reduced drag per unit mass. As a result, they can move through the fluid more quickly. Also, larger nanoparticles may have stronger inertia than smaller nanoparticles. When subjected to external forces, objects with a higher inertia need more force to alter their velocity. If the force driving the nanoparticles is constant, larger nanoparticles may experience less change in velocity in comparison with smaller nanoparticles. With the influence of these factors, larger nanoparticles gain higher velocities.

**Figure 14:**
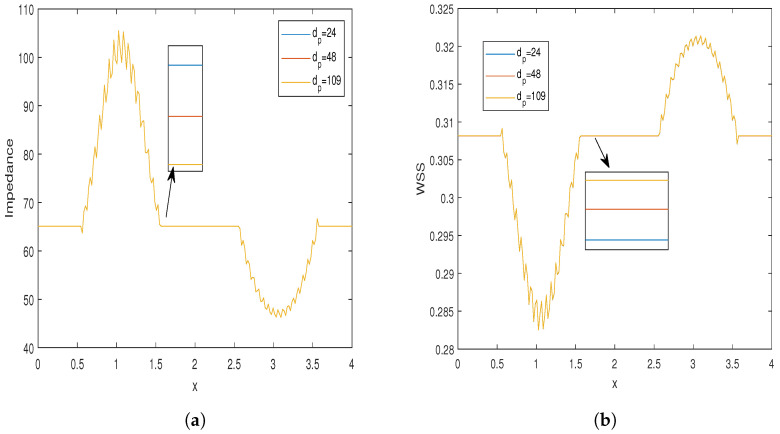
Variation in (**a**) impedance and (**b**) wall shear stress w.r.t. diameter of nanoparticles.

Variation in impedance to the flow concerning the diameter of $Fe_{3}O_{4}$ is shown in Figure 14a. A number of things, including the presence of materials like $Fe_{3}O_{4}$ (iron oxide) nanoparticles, can influence the blood’s flow rate. When we talk about the relationship between flow rate fluctuation and the volume percentage of $Fe_{3}O_{4}$ in blood flow, we’re probably talking about scenarios involving the introduction of nanoparticles into the bloodstream for magnetic drug targeting or magnetic resonance imaging (MRI) contrast agents. Because of their presence, an increase in the volume percentage of $Fe_{3}O_{4}$ nanoparticles in the blood may cause changes in blood viscosity. This might modify flow resistance, which would change the flow rate. Due to their magnetic properties, $Fe_{3}O_{4}$ nanoparticles may encounter magnetic forces that could affect how they are distributed throughout the bloodstream when exposed to an external magnetic field. This can have an impact on flow dynamics. Due to their decreased surface area-to-volume ratio, larger nanoparticles may interact with blood constituents like proteins and cells less frequently, which lowers impedance. In comparison to tiny nanoparticles, larger nanoparticles often agglomerate less in blood flow. Less agglomeration results in less blood flow obstruction and, as a result, reduced impedance. Compared to smaller nanoparticles, which may clump together more readily, larger nanoparticles may diffuse more uniformly in blood flow, improving dispersion and reducing resistance. Because larger nanoparticles may have a lower surface charge density than smaller ones, they may interact with charged blood components—such as ions and proteins—weakly, which lowers impedance. The variation in impedance to the flow with regard to the diameter of $Fe_{3}O_{4}$ is shown in Figure 14b. This shows that WSS in blood flow increases with increasing $Fe_{3}O_{4}$ nanoparticle diameter due to factors such as increased flow disruptions, blood viscosity, particle aggregation, greater particle collisions, bigger hydrodynamic diameters, and enhanced magnetic effects. These elements collaborate to increase resistance and resistance within the blood flow, resulting in increased shear stress on the arterial walls.

**Figure 15:**
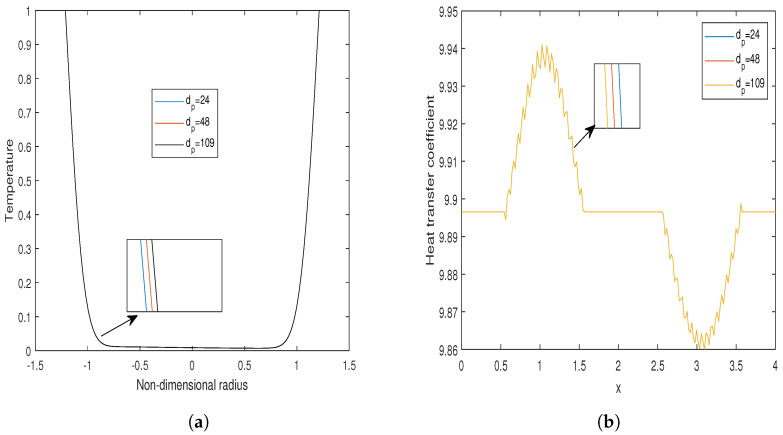
Variation in (**a**) nanofluid temperature and (**b**) heat transfer coefficient w.r.t. diameter of nanoparticles.

Figure 15a shows the increase in nanofluid temperature concerning the increment in diameter of the nanoparticle. Nanoparticles have increased thermal conductivity relative to the base fluid. As the diameter of $Fe_{3}O_{4}$ nanoparticles rises, so does their ability to transmit heat, resulting in improved heat transfer within the fluid. This can cause a spike in fluid temperature. Smaller nanoparticles often have a larger surface area-to-volume ratio. As the diameter rises, the ratio lowers. Larger nanoparticles, on the other hand, still have a huge surface area for interaction with the fluid, which could result in greater heat transfer. If the volume percentage of nanoparticles in the fluid remains constant while the diameter rises, more material will be spread in the fluid, potentially leading to increased heat transfer and therefore fluid temperature. Variation in the heat transfer coefficient concerning diameter of $Fe_{3}O_{4}$ is shown in Figure 15b. The reduction in surface-area-to-volume ratio, the reduction in Brownian motion, the lessening of thermal boundary layer disturbance, the increase in viscosity, the increase in aggregation, and the greater interfacial thermal conductivity are the main reasons why the heat transfer coefficient drops as nanoparticle diameter increases. These factors diminish heat transfer efficiency in nanofluids containing larger nanoparticles.

### 5.5. Variation w.r.t. Hematocrit Parameter and Radius of Curvature

**Figure 16:**
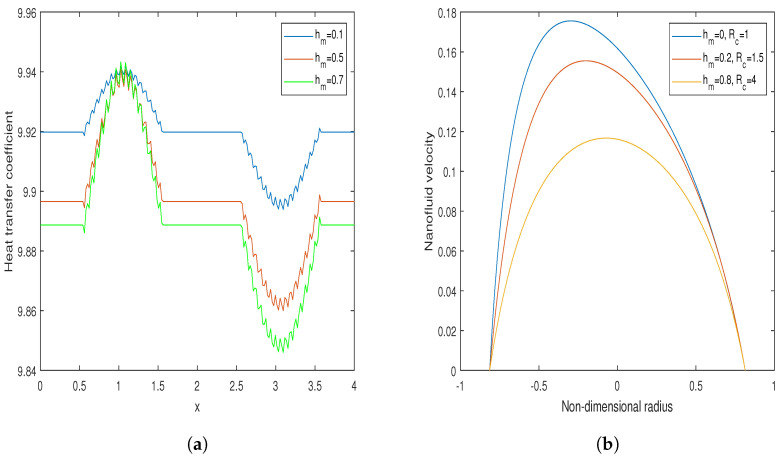
Variation of (**a**) heat transfer coefficient and (**b**) nanofluid velocity w.r.t. hematocrit parameter.

Figure 16a shows the variation in nanofluid velocity with hematocrit parameter and radius of curvature of the artery. Both the radius of curvature and the hematocrit level affect the nanoblood velocity in a curved artery. A higher hematocrit parameter enhances viscosity and reduces velocity overall, yet a shorter radius of curvature produces more complex secondary flows, which can drastically modify local velocities. However, the greater radius of curvature causes less disruption from secondary flows, resulting in a more predictable reduction in velocity and smoothing of the velocity profile. Figure 16b demonstrates the variation in heat transfer coefficient with the hematocrit parameter. Hematocrit can affect the heat transfer coefficient in blood flow variously. Hematocrit has a major impact on blood viscosity. Blood viscosity increases in conjunction with an increase in hematocrit. Blood flow is slowed by higher viscosity, which may have an impact on the convective heat transfer coefficient. Higher viscosity often causes the flow to become more sluggish, which lowers the convective heat transfer coefficient. In addition, blood’s thermal conductivity is also influenced by hematocrit. The total thermal conductivity of blood rises with hematocrit because plasma has a higher thermal conductivity than red blood cells, even though red blood cells have relatively low thermal conductivity. This may have an impact on blood flow’s general heat transmission properties. Hematocrit has an impact on the thickness of the boundary layer in close proximity to blood vessel walls. Changes in flow dynamics cause the boundary layer to thicken as hematocrit rises. Because it modifies the heat transfer mechanism close to the vessel walls, this larger boundary layer may have an impact on the convective heat transfer coefficient. Red blood cells make up the hematocrit or percentage of blood volume. Blood becomes more viscous and thickens as hematocrit rises. Increases in viscosity hinder a fluid’s capacity to transmit heat effectively. Viscosity is a measure of a fluid’s resistance to flow. Therefore, blood viscosity rises with hematocrit, decreasing the heat transfer coefficient in the process. A higher hematocrit can make blood flow less quickly. Convective heat transmission in the blood is lowered as a result of this drop in flow velocity. Reduced flow velocity leads to decreased convective heat transfer coefficients because heat transfer through convection is directly related to flow velocity.

### 5.6. Variation Concerning Eckert Number

**Figure 17:**
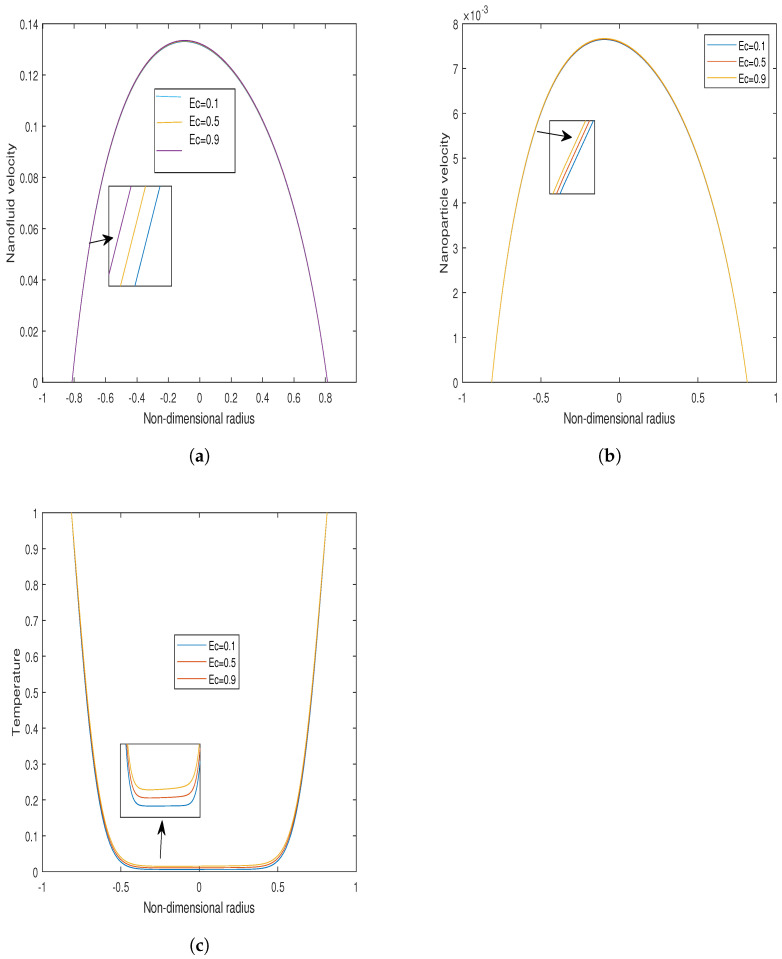
Variation of (**a**) nanofluid velocity, (**b**) nanoparticle velocity, and (**c**) nanofluid temperature w.r.t. Eckert number.

Figure 17a shows the increase in nanofluid velocity with the rise in the Eckert number. Nanofluids often have better thermal conductivity than base fluids. When the Eckert number (the ratio of kinetic energy to enthalpy) rises, thermal conductivity becomes more important. This increased thermal conductivity can improve heat transfer efficiency, allowing the fluid to absorb and release heat more effectively. As a result, the fluid temperature changes more quickly, causing convection currents and increasing fluid velocity. Figure 17b shows the increase in nanoparticle velocity with rise in Eckert no. As the Eckert number rises, thermal gradients in bloodstreams become more prominent (because of localized heating from extrinsic sources). Ferric oxide nanoparticles can absorb and transform energy into heat, causing localized hot regions in the blood. These regions of heat cause temperature gradients, which drive convection currents throughout the blood. Higher Eckert numbers augment this convective current, resulting in a faster velocity of nanoparticles. Hence, the velocity of Ferric oxide nanoparticles in the blood is enhanced with a rise in the Eckert number, owing to improved thermal effects such as increased heat transfer rates and stronger convective currents caused by thermal as well as magnetic variations produced by external sources of energy or fields. Figure 17c shows the increase in nanofluid temperature with the rise in Eckert number. When the Eckert number rises, it indicates that the fluid’s kinetic energy (due to motion) has become more important than the enthalpy change (which is related to thermal processes such as heating or cooling). This higher kinetic energy can cause more internal friction and disturbance inside the fluid, which in turn raises the rate of energy dissipation, as fluid motion at higher Eckert numbers causes increased frictional forces and internal energy dissipation. The conversion of kinetic energy (from fluid motion) to thermal energy (due to frictional heating and disturbance) generates an overall increase in fluid temperature.

**Figure 18:**
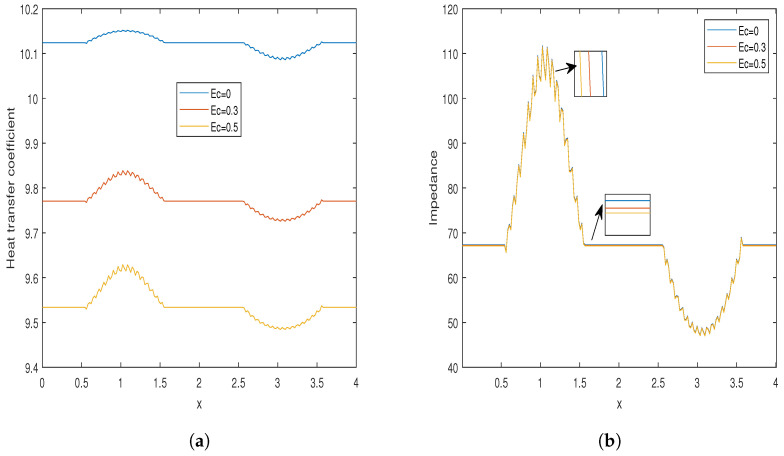
Variation of (**a**) heat transfer coefficient and, (**b**) impedance w.r.t. Eckert no.

Figure 18a depicts that as the Eckert number increases, there is a downfall in the heat transfer coefficient. The fluid heats as the Eckert number rises due to increased viscous dissipation, which also lowers the temperature gradient at the surface and the heat transfer coefficient. This effect is especially important in high-speed flows or flows that have substantial viscous effects when the fluid’s kinetic energy is greater than the temperature difference that drives heat transfer. The impedance to flow reduces as the Eckert number increases, owing to the effect of viscous dissipation, which raises fluid temperature, as can be seen in Figure 18b. This temperature increase frequently causes a drop in fluid viscosity, lessening internal friction as well as resistance to flow and so lowering total impedance.

### 5.7. Variation w.r.t. Electro-Osmotic Parameter

As per the model, the electro-osmotic force is mostly localized close to the artery wall, and an averaged continuum approximation of the EDL effect is used in the current model. The primary transport mechanism in arteries is still pressure-driven flow, and when electric fields are provided externally, the electro-osmotic contribution is regarded as a secondary coupled effect.

**Figure 19:**
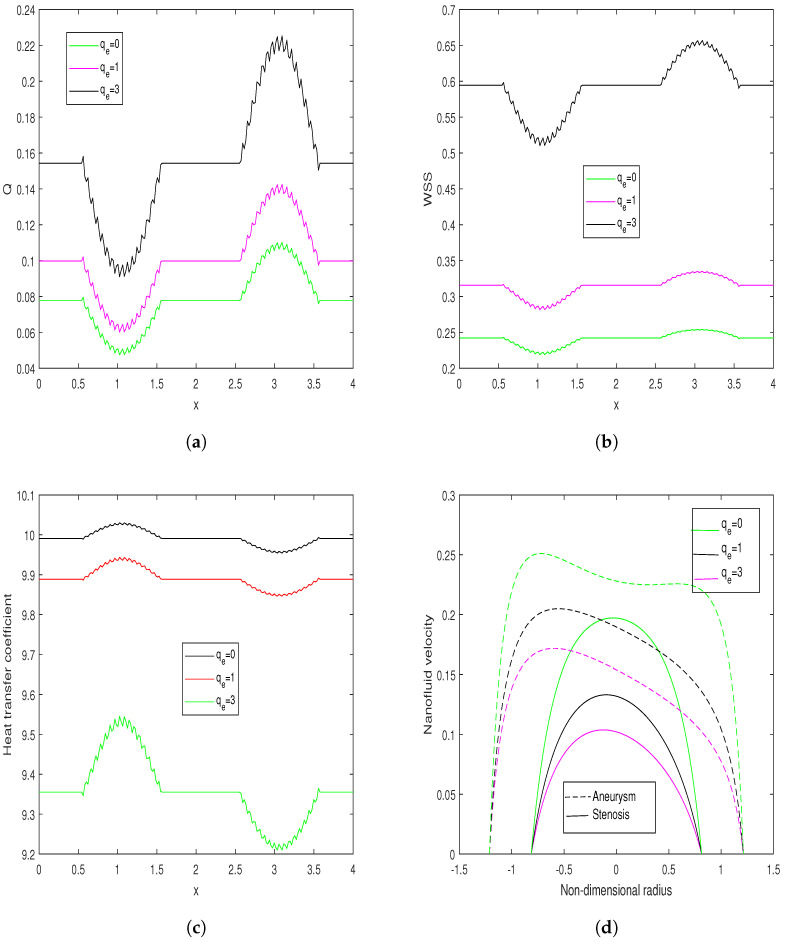
Variation of (**a**) flow rate, (**b**) wall shear stress, (**c**) heat transfer coefficient, and (**d**) nanofluid velocity w.r.t. electro-osmotic parameter.

Figure 19a depicts the variation in flow rate with respect to electro-osmotic parameters. The flow rate rises with a rise in the electro-osmotic parameter, which is directly connected to the forces acting on the blood in the presence of an electric field. A greater electro-osmotic parameter indicates a stronger force that drives due to either a larger zeta potential or a stronger applied electric field, both of which promote fluid movement and result in a higher flow rate. Figure 19b shows the variation in shear stress at the arterial wall with respect to electro-osmotic parameters. As the electro-osmotic parameter has a direct influence on electro-osmotic velocity, wall shear stress rises as it increases. Higher zeta potential or electric field intensity increases the fluid’s velocity near the wall, resulting in a steeper velocity gradient. This steeper gradient increases the wall shear stress. As the driving forces for electro-osmotic flow grow, so do the frictional forces imparted by the fluid on the arterial walls, resulting in higher wall shear stress. Figure 19c illustrates the effect of the electro-osmotic parameter on the heat transfer coefficient. The heat transfer coefficient decreases as the electro-osmotic parameter increases, owing to changes in velocity and temperature profiles caused by electro-osmotic flow. These changes result in a more uniform velocity profile, thicker boundary layers, and smaller temperature gradients near the arterial walls, all of which contribute to lesser convective heat transfer efficiency. Figure 19d represents the decrease in nanofluid velocity concerning increment in electro-osmotic parameter. The electrophoretic mobility rises proportionally to the electrokinetic parameter. This can induce a decrease in the net velocity of nanoparticles in a fluid because the electric field directs an increased portion of their motion rather than the fluid flow. Stronger electrokinetic parameters can result in more electrostatic interactions between nanoparticles and fluid molecules or other charged particles. These interactions can slow the passage of nanoparticles through the fluid by forcing them to bind to or repel from surrounding surfaces or particles, lowering their total velocity. Also, in a fluid, charged nanoparticles are surrounded by an electric double layer, which is made up of ions. Changes in electrokinetic factors can affect the thickness and qualities of this double layer. This, in turn, affects the drag force that the nanoparticles encounter as they move through the fluid. An increase in the electrokinetic parameter may result in a thicker double layer, which increases drag force and decreases nanoparticle velocity.

### 5.8. Velocity Contours

**Figure 20:**
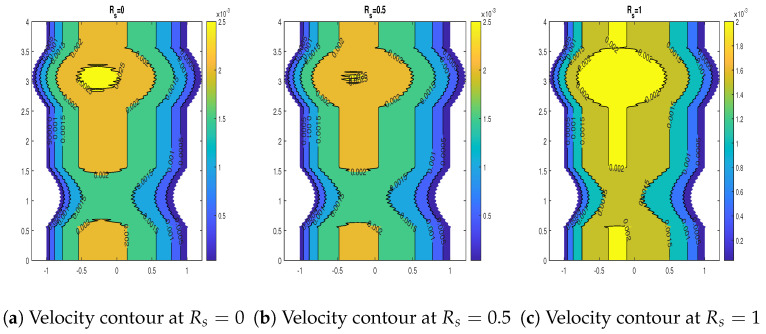
Variation of velocity contours w.r.t. various values of particle mass parameter.

Figure 20a–c depicts the variation in velocity contours concerning different values of the nanoparticle mass parameter. The given contour graphs show how the mass of magnetite nanoparticles affects flow velocity for a range of $R_{s}$ values. The relative mass concentration of nanoparticles in the blood is probably represented by the parameter $R_{s}$. For $R_{s}=0$ (nanoparticles not present) with clear central and boundary regions, the velocity field is comparatively uniform. A smoother flow distribution is shown by contour lines, indicating that there is less disruption when nanoparticles are not present. For a moderate concentration of nanoparticles ($R_{s}=0.5$), there is a small expansion of the core high-velocity zone. The increasingly intricate contour lines imply that the flow is being impacted by the nanoparticles. Nanoparticle-induced increased viscosity probably amplifies shear effects, changing the distribution of blood velocity. For $R_{s}=1$ (higher concentration of nanoparticles), the dominance of the high-velocity area suggests that the presence of nanoparticles has a major impact on flow characteristics. As the contour density rises, the velocity changes become more pronounced. The effective viscosity of blood is probably increased by higher concentrations of magnetite nanoparticles, which results in increased resistance and velocity redistribution. This can be physically interpreted as follows. Blood’s density and viscosity are changed by the magnetite nanoparticles, which has an immediate impact on velocity profiles. Nanoparticles have little effect and flow is nearly normal at low concentrations (small $R_{s}$). Due to modifications in fluid characteristics, nanoparticles begin to affect velocity at moderate concentrations (medium $R_{s}$). The flow undergoes notable changes at high concentrations (large $R_{s}$), exhibiting steeper gradients and a wider high-velocity area.

**Figure 21:**
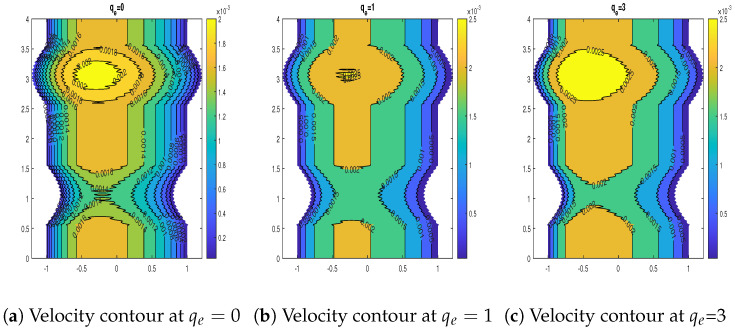
Variation in velocity contours w.r.t. various values of electro-osmotic parameters.

The given subfigures display blood flow velocity contours for various electro-osmotic parameter ($q_{e}$) values. For $q_{e}=0$, the Figure 21a represents the situation in which the electro-osmotic effect is absent. The pressure gradient and other hydrodynamic parameters primarily affect the velocity distribution. Lower velocities are found close to the edges, whereas the maximum velocity zone (yellow) is concentrated in the channel’s center. A symmetrical structure suggests a pressure-driven flow without further electrokinetic influence. For $q_{e}=1$ (Figure 21b), the flow starts to be affected by electro-osmotic processes. An increase in velocity caused by electrokinetic force is suggested by the minor expansion of the high-velocity zone near the middle. In contrast to $q_{e}=0$, velocity contours become more dispersed, signifying a change in flow structure. Peak velocity increases noticeably (yellow patches become more visible). For the value $q_{e}=3$ (Figure 21c), the velocity distribution is dramatically changed by a stronger electro-osmotic action. The yellow, high-velocity area gets bigger and more noticeable. Near the boundary, the velocity gradient shifts, indicating that fluid motion is being enhanced by electro-osmotic forces. The high-velocity zone is stretched more clearly in contours, suggesting that electro-osmotic effects outweigh pressure-driven effects. Overall findings show fluid velocity is improved by increasing, particularly in the core area. By redistributing velocity, electro-osmotic effects improve the uniformity of the flow. The contour patterns point to changed velocity gradients close to the walls and an increase in flow rate.

**Figure 22:**
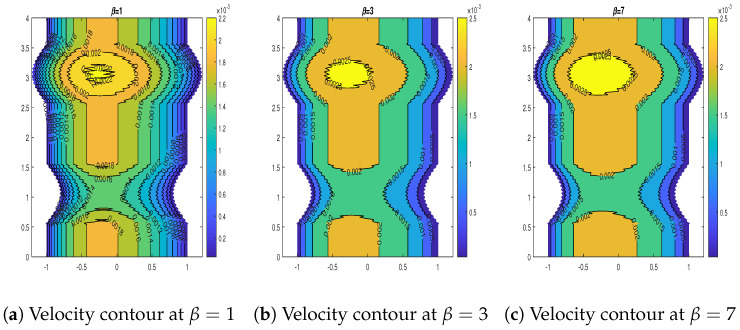
Variation in velocity contours w.r.t. various values of Casson parameter.

In the above figures, the velocity contour fluctuation is displayed for various Casson parameter (β) values. The velocity distribution is impacted by the variation in the following ways: For β=1 (Figure 22a), the yellow area in the velocity distribution indicates a comparatively lower maximum velocity. Higher shear stress and flow resistance are shown by the closely spaced contour lines. The less expanded core high-velocity zone suggests that the fluid’s non-Newtonian nature caused by a lower β dominates and limits flow augmentation. For β=3 (Figure 22b), the velocity magnitudes have grown, with a larger yellow zone in the middle, compared to β=1. A decrease in shear stress is suggested by the contour lines’ increased structure and minor spacing. As β rises, the flow resembles a Newtonian fluid, improving the velocity distribution. For β=7 (Figure 22c), the greater high-velocity (yellow) zone indicates that the maximum velocity has been further increased. There is less resistance in the flow, as indicated by the more evenly spaced contour lines. The velocity contours are more flattened in the vertical direction, implying that higher β promotes more equal velocity dispersion. The velocity increases as the Casson parameter (β) increases. The velocity contours become more consistent as the flow resistance drops.

**Figure 23:**
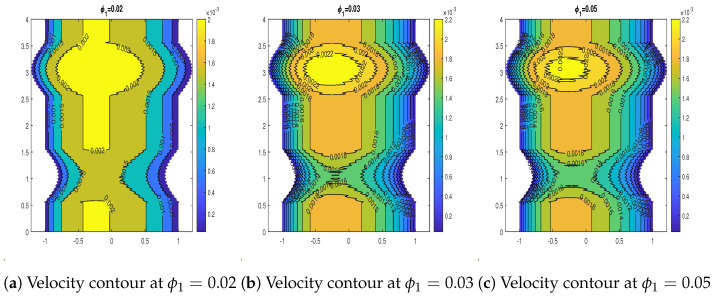
Variation of velocity contours w.r.t. various values of volume fraction of nanoparticles.

Relevant to the targeted use of medications, the above figure shows how blood flow velocity contours vary for various $Fe_{3}O_{4}$ nanoparticle volume fractions ($\phi_{1}$). For $\phi_{1}=0.02$ (Figure 23a), the velocity distribution exhibits wider core regions with greater velocity (yellow regions) for a lower-volume percentage of $Fe_{3}O_{4}$ nanoparticles. The moderate velocity gradients surrounding obstructions or constrictions suggest that there are comparatively fewer interactions between blood flow and nanoparticles. A smoother velocity transition is suggested by the more evenly spaced contours. For $\phi_{1}=0.03$ (Figure 23b), the velocity contours become increasingly compact as the volume fraction rises, particularly in the vicinity of the restricted areas. In certain places, the velocity has increased, which could mean that the nanoparticles and blood flow are interacting more effectively. Sharper gradients are the result of higher flow resistance brought on by a higher particle concentration. And for $\phi_{1}=0.05$ (Figure 23c), the velocity distribution displays even more compact outlines at the maximum volume fraction displayed, especially in high-velocity areas. A more noticeable velocity fluctuation results from $Fe_{3}O_{4}$ nanoparticles’ increased viscosity. Stronger shear effects are visible, which may have an impact on how the medication disperses in blood flow. By raising the volume fraction of $Fe_{3}O_{4}$ nanoparticles change the velocity profile. Steeper velocity transitions and more confined high-velocity areas are the results of higher volume fractions. Because it influences how well the nanoparticles move throughout the bloodstream, this characteristic is essential for targeted medication delivery.

## 6. Conclusions

The current study investigated the impact of heat transfer and the incorporation of $Fe_{3}O_{4}$ nanoparticles on blood flow through a curved channel with moderate stenosis and aneurysm conditions, characterized by variable viscosities. This concept utilizes nanoparticles to enhance the delivery of medicine in a narrow, curved artery. The graphs depict the axial change in blood velocity, temperature, flow rate, impedance, Nusselt number, and wall shear stress variation for a variety of emergent parameter values. Biologists can use the mathematical analysis from the current model to reduce the risk of lipid, fat, and cholesterol accumulation in the arteries. By recognizing the range of symptoms, they can also utilize it to forecast the likelihood of cardiovascular disease and the present condition of anomalies. They may then suggest diagnostic procedures and treatment based on the risk of arterial spasms and aneurysm rupture. The results can also be applied to the design and analysis of nano-drug delivery systems, the anticancer drug business, and biomedical devices for highly promising therapeutic modalities. The following list summarizes the significant findings:

- Both nanofluid and nanoparticle velocity increase as the Casson parameter increases.
- As the volume fraction of $Fe_{3}O_{4}$ increases, both nanofluid and nanoparticle velocity decrease.
- Temperature of nanofluid also increases with the increment in the volume fraction of nanoparticles.
- As the particle mass parameter rises, there is a decrease in nanoparticle and nanofluid velocity.
- Velocity of nanofluid decreases as the electro-osmotic parameter increases.
- As the diameter of the nanoparticle increases, nanofluid and nanoparticle velocity and nanofluid temperature increase.
- As Eckert number increases, the velocity and temperature of nanofluid increase.
- The heat transfer coefficient decreases as the hematocrit parameter increases.
- With the increment in the volume fraction of the nanoparticles, wall shear stress increases, whereas flow rate and heat transfer coefficient decrease.
- Wall shear stress increases with the increment in the diameter of the nanoparticle, whereas the reverse effect can be seen with the increment in the particle mass parameter.
- As the particle mass parameter increases, there is a small expansion of the core high velocity zone in the velocity contours.

Superparamagnetic property of $Fe_{3}O_{4}$ lowers the danger of embolism or vessel obstruction and stops blood particles from aggregating. Site-specific medication administration is made possible by an externally applied magnetic field, which also improves the drug concentration at the targeted site and minimizes adverse effects on healthy tissues. Biocompatible coatings such as dextran, PEG, or silica can be used to functionalize $Fe_{3}O_{4}$ nanoparticles, which have a huge surface area. Magnetite nanoparticles can produce localized heat when exposed to an alternating magnetic field. In addition to chemotherapy, this can be utilized to improve medication release and destroy cancer cells (thermotherapy). Magnetite nanoparticles are biocompatible and non-toxic when appropriately coated. Through the body’s regular iron metabolic pathways, they can be broken down and eliminated.

## 7. Future Research Prospective

The following is a summary of potential future research directions for the current study:

- Three-dimensional flow modeling: Since the current work considers axisymmetric flow, future research may take into account fully three-dimensional modeling to simulate asymmetric recirculation zones, secondary flow structures, and Dean vortices in curved stenosed and aneurysmal arteries.
- Tracking of Lagrangian nanoparticle: To investigate trajectories of individual nanoparticle, residence duration, targeting efficiency, and deposition in diseased arteries, future research may use Lagrangian particle-tracking techniques rather than the present averaged Eulerian methodology.
- More accurate electrokinetic modeling: The current model makes use of the Debye–Hückel approximation and constant zeta potential assumptions. Future research might focus on realistic electrical double layer behavior under physiological settings, changeable surface charge, and nonlinear Poisson–Boltzmann equations.
- Additional particle forces: In order to improve the prediction of nanoparticle transport, future models may include Brownian motion, Basset history force, virtual mass force, Saffman lift force, thermophoresis, and particle–particle interactions.
- Patient-specific arterial geometry: The current artery geometry is hypothetical. Future research can use CT/MRI to simulate blood flow and nanoparticle transportation in individual-specific arterial configurations.
- Non-Newtonian blood rheology: To more accurately depict shear-thinning behavior, viscoelasticity, and red blood cell interactions, more sophisticated constitutive models could be taken into consideration.
- Pulsatile and transitory physiological conditions: Future research may examine fluid–structure interaction, vascular wall flexibility, and completely time-dependent heart pulsation effects in electro-osmotic MHD blood flow.
- Targeted drug delivery applications: Future research may examine localized therapeutic delivery in damaged arteries, regulated nanoparticle deposition, magnetic drug targeting, and the treatment of hyperthermia. To maximize biomedical transport performance, the effects of various nanoparticle materials, shape, concentrations, and hybrid nanofluids may be examined.
- To further determine the boundaries of the application of electrokinetic models in biological flows, future research may systematically evaluate electro-osmotic impacts on microchannels and arteries.

## Figures and Tables

**Table 1:** Dimensionless parameters.

$r=\bar{r}R_{0}$	$x=\bar{x}\lambda_{i}$	$u=\frac{\bar{u}\delta^{*}u_{0}}{\lambda_{i}}$	$v=\bar{v}u_{0}$
$T=T_{0}+\theta \left(T_{w}-T_{0}\right)$	$t=\frac{\bar{t}R_{0}}{u_{0}}$	$R^{*}=R_{c}R_{0}$	$p=\frac{\bar{p}\mu_{0}u_{0}\lambda_{i}}{R_{0}^{2}}$
$\delta^{*}=\delta R_{0}$	$B_{0}^{2}=\frac{\mu_{0}M^{2}}{\sigma_{f}R_{0}^{2}}$	$Gr=\frac{g{\left(\rho \eta \right)}_{f}R_{0}^{2}\left(T_{w}-T_{0}\right)}{\mu_{0}u_{0}}$	$Br=EcPr=\frac{\mu_{0}u_{0}^{2}}{k_{f}\left(T_{w}-T_{0}\right)}$
$Ec=\frac{u_{0}^{2}}{{\left(C_{p}\right)}_{f}\left(T_{w}-T_{0}\right)}$	$Pr=\frac{\mu_{0}{\left(C_{p}\right)}_{f}}{k_{f}}$	$Re=\frac{u_{0}\rho_{f}R_{0}}{\mu_{0}}$	$U_{h_{0}s}=-\frac{\zeta_{0}\Upsilon E_{0}}{\mu_{0}u_{0}}$
$S_{j}=\frac{\sigma_{f}R_{0}^{2}E_{0}^{2}}{k_{f}\left(T_{w}-T_{0}\right)}$	$\epsilon =\frac{R_{0}}{\lambda_{i}}$	${\bar{d}}_{i}=\frac{d_{i}}{\lambda_{i}}$	$q_{e}=\frac{q_{m}}{R_{0}}$
$c_{1}=\frac{2\pi R_{0}\omega_{p}}{u_{0}}$	$B_{1}=\frac{A_{0}R_{0}^{2}}{\mu_{0}u_{0}}$	$e=\frac{A_{1}}{A_{0}}$	$\alpha =\frac{\alpha^{*}\lambda_{i}}{R_{0}}$

**Table 2:** Thermophysical properties of blood and *Fe*_3_*O*_4_ nanoparticles.

Property	Fe_3_O_4_	Blood
Thermal Conductivity [*K* (W/mK)]	6	0.492
Electrical Conductivity [σ (S/m)]	$2.5\times 10^{4}$	0.667
Density [ρ (kg/m^3^)]	5200	1063
Thermal Expansion Coefficient [$\eta \times 10^{-5}$ ($K^{-1}$)]	1.3	0.18
Heat Capacitance [$C_{p}$ (J/kgK)]	670	3594

**Table 3:** Physical parameter values.

Parameters	Range	Sources
Magnetic Number	0–4	[49]
Hematocrit Parameter	0–2	[3]
Thermal Grashof Number	0–6	[50]
Prandtl Number	0–4	[19]
Reynold’s Number	1–10	[51]
Eckert Number	0–1	[52]

**Table 4:** Default values of some physical parameters.

Parameters	$B_{1}$	$c_{1}$	$\phi_{1}$	*e*	$M_{a}$	$d_{p}$	$U_{\mathrm{hs}}$	$R_{c}$	$R_{s}$	$T_{f}$	$T_{\mathrm{fr}}$
**Values**	1.41	2π	0.02	0.2	64.5	23	2	3	1	310	272.6

## Data Availability

The authors affirm that all data supporting the conclusions of this study are comprehensively presented within the article.

## Abbreviations

The following abbreviations are used in this manuscript:

*r*	Radial direction
*x*	Axial direction
*t*	Time
*u*	Radial Velocity component
*v*	Axial velocity component
$u_{0}$	Reference velocity
$R^{*}$	Radius of the curved channel
$R_{0}$	Radius of normal artery
$R_{c}$	Dimensionless radius of curvature of the artery
*T*	Temperature of the base fluid
$T_{0}$	Reference temperature
$T_{w}$	Temperature at the wall
$C_{0}$	Average concentration
$q_{e}$	Electro-osmotic parameter
*Q*	Volumetric flow Rate
$f_{p}$	Heart pulse frequency
$f_{b}$	Frequency of body acceleration
$S_{z}$	Term related to Joule heating
$U_{hs}$	Helmholtz-Smoluchowski velocity
*g*	Acceleration by gravity
$A_{1}$	Amplitude of pulsatile component
$A_{0}$	Amplitude of pressure gradient
$B_{1}$	Dimensionless pulsatile constant
*C*	Concentration of the base fluid
$C_{p}$	Specific heat at constant pressure
*P*	Pressure
$B_{1}$	Pressure gradient parameter
$K_{f}$	Thermal conductivity
*B*	Uniform Magnetic Field
$D_{m}$	Diffusivity constant
*e*	Systolic to diastolic pressure ratio
${\left(\rho C_{p}\right)}_{nf}$	Heat capacitance of nanofluid
θ	Non-dimensional temperature
$\rho_{nf}$	Density of nanofluid
$\phi_{1}$	Nanoparticle’s Concentration
η	Thermal expansion coefficient
$M^{2}$	Hartmann Number
Re	Reynold’s Number
Gr	Thermal Grashof Number
Sc	Schmidt Number
Pr	Prandtl Number
$E_{1}$	Electric field parameter
λ	Resistance Impedance
$\mu_{f}$	Blood’s viscosity
σ	Electrical conductivity
$\tau_{w}$	Shear stress at the wall
δ	Stenosis depth
ξ	Chemical reaction parameter
$\omega_{p}$	Circular frequency
$\mu_{0}$	Reference viscosity
